# Site-specific gene expression profiling as a novel strategy for unravelling keloid disease pathobiology

**DOI:** 10.1371/journal.pone.0172955

**Published:** 2017-03-03

**Authors:** N. Jumper, T. Hodgkinson, R. Paus, A. Bayat

**Affiliations:** 1 Plastic and Reconstructive Surgery Research, University of Manchester, Oxford Rd, Manchester, United Kingdom; 2 Centre for Tissue Injury and Repair, University of Manchester, and MAHSC, Manchester, United Kingdom; 3 Centre for Dermatology Research, University of Manchester, and MAHSC, Manchester, United Kingdom; Sinai Hospital, UNITED STATES

## Abstract

Keloid disease (KD) is a fibroproliferative cutaneous tumour characterised by heterogeneity, excess collagen deposition and aggressive local invasion. Lack of a validated animal model and resistance to a multitude of current therapies has resulted in unsatisfactory clinical outcomes of KD management. In order to address KD from a new perspective, we applied for the first time a site-specific *in situ* microdissection and gene expression profiling approach, through combined laser capture microdissection and transcriptomic array. The aim here was to analyse the utility of this approach compared with established methods of investigation, including whole tissue biopsy and monolayer cell culture techniques. This study was designed to approach KD from a hypothesis-free and compartment-specific angle, using state-of-the-art microdissection and gene expression profiling technology. We sought to characterise expression differences between specific keloid lesional sites and elucidate potential contributions of significantly dysregulated genes to mechanisms underlying keloid pathobiology, thus informing future explorative research into KD. Here, we highlight the advantages of our *in situ* microdissection strategy in generating expression data with improved sensitivity and accuracy over traditional methods. This methodological approach supports an active role for the epidermis in the pathogenesis of KD through identification of genes and upstream regulators implicated in epithelial-mesenchymal transition, inflammation and immune modulation. We describe dermal expression patterns crucial to collagen deposition that are associated with TGFβ-mediated signalling, which have not previously been examined in KD. Additionally, this study supports the previously proposed presence of a cancer-like stem cell population in KD and explores the possible contribution of gene dysregulation to the resistance of KD to conventional therapy. Through this innovative *in situ* microdissection gene profiling approach, we provide better-defined gene signatures of distinct KD regions, thereby addressing KD heterogeneity, facilitating differential diagnosis with other cutaneous fibroses via transcriptional fingerprinting, and highlighting key areas for future KD research.

## Introduction

Keloid disease (KD) is a fibroproliferative cutaneous tumour of ill-defined pathogenesis characterised by clinical, behavioural and histological heterogeneity [1]. Keloid scars, consisting mostly of hyalinised collagen bundles, spread beyond the boundaries of the original wound resulting in “claw-like” or “cheloide” invasions into adjacent normal skin. KD research has been hindered by lack of a validated animal model, a paucity of tissue for experimentation secondary to ethical concerns over high rates of recurrence following excision [2] and both inter-patient and inter-lesional heterogeneity [3]. The multitude of available therapies, including first-line non-invasive treatments (compression garments, physiotherapy, camouflage), second-line treatments such as corticosteroid injection or cryotherapy and more extreme experimental agents including chemotherapy and radiotherapy, indicate the lack a gold standard effective treatment option for KD [4]. In an effort to overcome this, our previous studies including others have considered KD in terms of different lesional sites within the keloid scar: intralesional (centre), perilesional (margin) and extralesional (adjacent normal skin) [5].

The evidence for site-specific KD is threefold. Macroscopically, the centre is often pale soft and shrunken when compared with the raised erythematous margin. Microscopically, there are differences with respect to epidermal thickness, inflammatory infiltrate, collagen ratios and cellularity [6, 7]. Finally, on a molecular level, these sites have been shown to differ with regard to cell cycle phase and apoptotic factor expression [8]. While this site-specific approach has highlighted the diversity within KD, the use of whole tissue biopsy and monolayer culture fail to accurately reflect the *in situ* expression of this unique 3D microenvironment.

**Fig 1:**
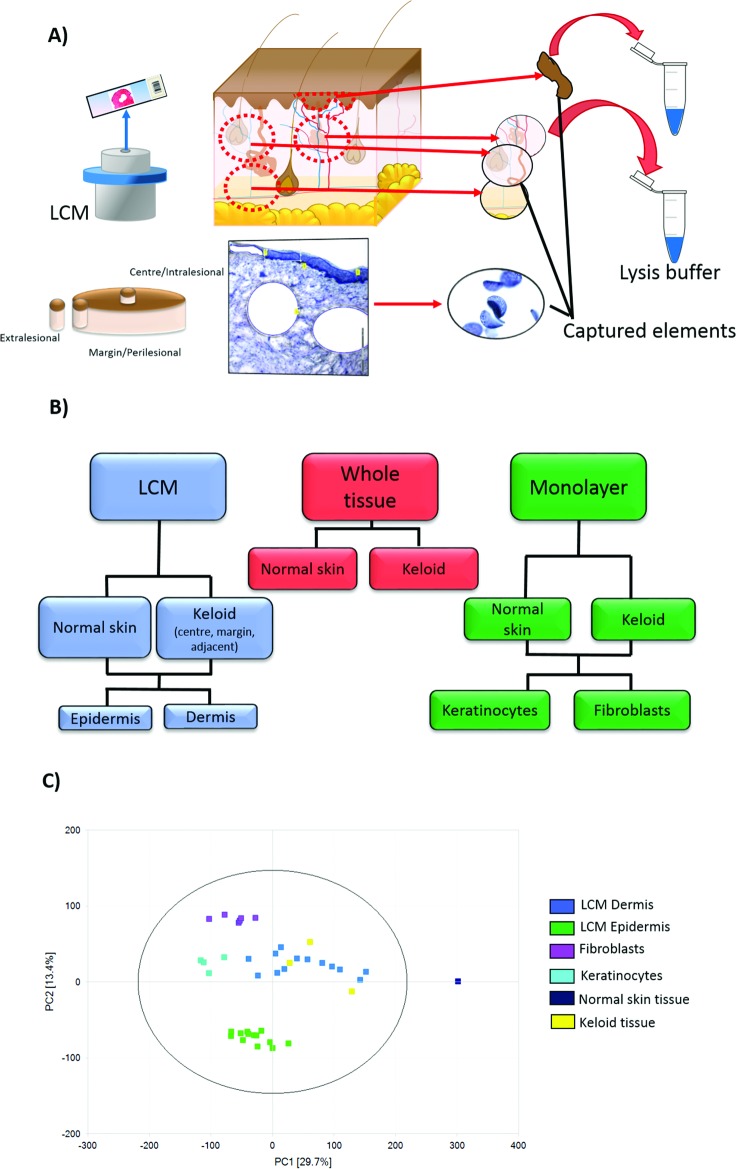
Experimental approaches for the comparison of site-specific keloid disease with normal skin. A) Schematic diagram demonstrating laser capture microdissection (LCM) of epidermis and dermis for each of the shown keloid biopsy sites, centre (intralesional), margin (perilesional) and keloid-adjacent normal skin (extralesional). LCM was performed for keloid sites and normal skin. As shown, the elements pertaining to portions of each compartment (epidermis separate to dermis) were delineated, cut using ultraviolet (UV) laser and catapulted into the cap of an overhanging tube, where images confirmed their presence. This was then immersed in lysis buffer and stored at -80°C. B) The three methods of experimental technique used to compare keloid with normal skin: LCM, whole tissue biopsy and 2D monolayer cell culture. C) Principal component analysis (PCA) plot for the gene expression derived from experimental approaches described above. The epidermal and dermal samples are evident as separate clusters as are the laser captured material and the monolayer culture samples.

Therefore, in addition to site-specific, the second aspect of our approach was to examine *in situ* signalling. We achieved this by combining laser capture microdissection (LCM) and transcriptomic array profiling (**Fig 1A**). To date, LCM has played a limited role in cutaneous wound healing but given its success both in other areas of fibrosis research as well as benign and malignant dermatological conditions [9–11], we felt it was an ideal methodological platform for application to KD. This approach also allowed us to focus on individual expression in different regions of the keloid scar without the need for “averaging out” of signals commonly consequential to whole tissue biopsy analysis or the altered expression that can result from the *in vitro* environment of monolayer cell culture [12].

The overall aim of this study was not to validate specific theories but to apply an innovative hypothesis-free and compartment-specific approach to KD. Thus, the first aim was to compare this combined LCM and microarray (*in situ*) approach to both whole tissue biopsy and monolayer culture methods of analysing gene expression (**Fig 1B**). The second aim was to enrich the data in terms of gene ontology, in an effort to explore the biological processes within different lesional sites of the keloid scar. Finally, we aimed to examine differentially expressed genes (DEG) from each site of both epidermis and dermis compared with micro-dissected normal skin (NS), to identify pathways for potential diagnostic and therapeutic exploitation.

We show that our *in situ* approach most accurately reflects the *in vivo* environment without missing functionally important DEG through dilution and averaging out. These DEG indicate an activated epidermis with a potential for epithelial-mesenchymal transition (EMT) and expose dermal collagen-promoting molecules of which *TGF*β is an integral component but which remain overlooked in KD. The sensitivity of this technique allowed us to unveil another piece of the complex inflammatory network contributing to KD, unravel some of the elements contributing to therapeutic resistance and strengthen the argument for a stem cell population in KD [13].

## Materials and methods

### Study approval

Keloid and NS tissue were harvested at the time of surgery following full verbal and written consent obtained in accordance with the Declaration of Helsinki. The North West Research Ethics Committee (NorthWest of England, UK) approved this specific study (ethical Reference number. 11/NW/0683). In total there were 8 of each NS and keloid tissue donors used for microarray, with additional samples included for supporting data (**S1 Table**). The scar was considered to be keloid if it fulfilled the following criteria: growth beyond the boundaries of the original wound, failure to regress with time, present for at least one year and lesions that would recur with excision alone [14, 15].

### Tissue processing

Tissue biopsies were taken from keloid scar centre, margin and extralesional sites (**Fig 1A**). NS biopsies were from patients undergoing routine non-oncologic elective surgery. Biopsies were immediately preserved in either RNA stabilisation solution (RNA*later*®, Life technologies Ltd, Paisley, UK) or 10% (v/v) neutral buffered formalin (Sigma-Aldrich, UK). The RNA stabilised samples were OCT (optimum cutting temperature)-embedded (CellPath, UK) and snap frozen before being stored at -80°C.

### Laser capture microdissection

Serial 8μm cryosections (Leica CM3050S, UK) of OCT-embedded keloid and NS samples were cut onto specialised polyethylene naphthalate (PEN) membrane slides (Carl Zeiss, UK). To differentiate epidermis from dermis, whilst preserving tissue RNA integrity, a rapid staining protocol was performed (LCM Staining Kit, Ambion, Austin TX, USA) according to the manufacturer’s instructions [16, 17]. Using a P.A.L.M. LCM microscope (Carl Zeiss MicroBeam 4.2, Germany) epidermis and dermis of each sample was laser cut and catapulted away from the slide into separate overhanging microtube caps (AdhesiveCap 200 Opaque, Carl Zeiss Microscopy Ltd, Cambridge, UK). Multiple ‘elements’ were captured from each tissue section of least three sequential sections from each patient, ensuring adequate biological representation. The captured tissue was mixed with lysis buffer (Buffer RLT with 1% 2-mercaptoethanol, RNeasy Micro Kit, Qiagen, UK) and stored at -80°C until extraction according to manufacturer’s instructions (RNeasy Micro Kit, Qiagen, UK). Following extraction, the samples were again stored at -80°C [18].

### RNA amplification and microarray

Extracted RNA was amplified using the Ovation® Pico WTA system v2 kit (NuGen Technologies, USA) and purified with QIAquick PCR purification kit (Qiagen, UK), according to manufacturer’s instructions. Prior to microarray, RNA quantity was estimated using a microvolume spectrophotometer (ThermoScientific NanoDrop 2000 UV-vis, USA). Agilent SureTag DNA labelling and hybridisation kit were used according to manufacturer’s instructions and slides (SurePrint G3 Human GE 8x60K V2, Agilent Technologies, USA) scanned using an Agilent Microarray Scanner G2505c [19–21].

### Quantitative Real-Time Polymerase Chain Reaction (qRT-PCR)

qRT-PCR was performed using the Lightcycler® 480 II platform (Roche Diagnostics, UK) as previously described [22]. A final reaction volume of 10μl contained normalised cDNA, LightCycler®480 probes master mix, forward and reverse primers, nuclease-free water (Qiagen, UK) and the associated probe from the Universal Probe Library (Roche, UK). Reactions were performed in triplicate with two house-keeping genes (RPL32 and GAPDH) for relative quantification. Amplified targets were analysed using the Lightcycler® II software (1.5.0 SP3, Roche, UK).

### Cell culture

Primary keratinocytes and fibroblasts were established as previously described [22, 23]. In brief, tissue was cut and incubated in Dispase II (10mg/ml; Roche Diagnostics, UK) at 37°C. The epidermis was stripped, diced and incubated in TrypLE™Express (ThermoFisher Scientific, USA) with serum-free keratinocyte medium (Epilife®, Invitrogen Life Technologies, ThermoFisher Scientific, USA) for one hour before neutralising, centrifugation and dispersion into T25 flasks. The dermis was further incubated in collagenase before adding to complete DMEM and grown in flasks. Medium was changed every 48hrs until confluent. Passages 1–3 were used. Cells were lysed and RNA extracted using Qiagen RNeasy Micro Kit, according to manufacturer’s instructions.

### Statistical analysis

Data was extracted from the raw files and initial microarray analysis performed using Array studio v7.2 (OmicSoft Corporation, USA) and the data quantile normalised. A linear model was then fitted to the log_2_ transformed data for which both p-value and False Discovery Rate (FDR), controlled for using Benjamini-Hochberg method, were calculated for each group comparison [24]. Least squared means (LS means) and 95% confidence interval (where n>1) were outputted for each group. The data was then filtered using the following criteria: maximum median signal intensity >8 (leaving 46802 probe sets), p-value <0.05, fold change >2 and for individual genes of interest, q-value < 0.05 (**S3 and S4 Figs**). Lists of DEG were loaded into Ingenuity Pathway Analysis (IPA, Qiagen). IPA was chosen as the primary mode of enrichment analysis as all pathways, ontologies and interactions are manually curated and have supporting literature data behind them, thus providing a very robust and standardised platform for interpreting differential gene lists from transcriptomic studies. A full table of expanded gene names for each symbol discussed below can be found in **S2 Table**.

For qRT-PCR, expression was normalised against internal controls and ΔΔC_T_ calculated. Statistical analysis was performed using Student’s *t*-test and one way ANOVA with Tukey post-hoc correction (SPSS, IBM), where p-value <0.05 was considered significant [25]. Data are represented as mean ± SEM.

## Results & discussion

### Microarray analysis reveals variable differential gene expression based on experimental approach

Initial analysis was conducted to define site-specific gene expression, determine relationships between experimental approaches and establish networks based on correlation clustering. We compared gene expression for both epidermis and dermis between different sites within the keloid lesion, based on their expression difference over their NS epidermal and dermal counterpart. Additionally, we analysed whole tissue biopsy and monolayer culture (keratinocyte and fibroblast) expression for both KD and NS. The number of significant DEG within these comparative groups as well as their direction of change is shown in **Table 1**.

**Table 1:** Number of significant differentially expressed genes within each comparative microarray group (filtered for fold change > 2 and p-value < 0.05).

Harvest method	Comparison	Total sig. changes	Up	Down	FDR-corrected (q < 0.5)
**LCM**	*Keloid centre vs normal epidermis*	1165	591	574	18
**LCM**	*Keloid margin vs normal epidermis*	911	562	349	11
**LCM**	*Keloid extralesional vs normal epidermis*	1425	608	817	28
**LCM**	*Keloid centre vs normal dermis*	3640	1795	1845	1085
**LCM**	*Keloid margin vs normal dermis*	3818	1852	1966	882
**LCM**	*Keloid extralesional vs normal dermis*	3313	1549	1765	423
**Monolayer**	Keloid vs normal keratinocytes*	356	207	149	21
**Monolayer**	*Keloid vs normal fibroblasts*	247	146	101	3
**Whole tissue**	Keloid vs normal skin*	12527	7583	4944	9076

FDR, false discovery rate; LCM, laser capture microdissection.

*keloid keratinocyte and whole tissue normal skin biopsy *n* = 1.

Principal component analysis (PCA) was used to assess technical variability in the microarray QC metrics; the expected 5% of samples (2/40) lay outside the 95% confidence interval but were from two separate donors and therefore not excluded. Following quantile normalisation [26], probe sets were filtered by calculating maximum group median and removed if minimum signal intensity was < 8 (on log_2_ scale), leaving 24,228 probes (approximately 40%). PCA was employed to ascertain relationships between sample groups and compare variability between replicate arrays and experimental conditions [27].

This plot indicated the most significant variability existed between different cell types, that is epidermis and dermis or keratinocyte and fibroblast, which fell into separate clusters (X-axis) but variability was also found between the *in situ* micro-dissected cell layer and it’s *in vitro* monolayer culture equivalent i.e. epidermis and keratinocyte (Y-axis) (**Fig 1C**). Some of this variability could be attributed to the differentiation state of keratinocytes. Additionally, while keratinocytes and fibroblasts constitute the major cell type in the epidermis and dermis respectively, the *in situ* tissue layer will comprise additional cells that contribute to expression. Therefore, as expected, the epidermal layer and dermal layer, as well as their constituent cells, differed in their expression profiles. Interestingly however, this analysis also indicated there was differential gene expression dependent on the experimental approach, such that keratinocyte expression (derived from monolayer cell culture) differed from that of the laser-captured *in situ* epidermal expression. The same was true for the culture-derived fibroblasts and micro-dissected dermis.

Using weight gene correlation network analysis (WGCNA), a soft threshold was established and the microarray data was clustered into an eigengene networks, thereby allowing gene ontology enrichment based on consensus modules. This identified three key modules where KD expression diverged most significantly from that of NS. Transforming growth factor beta (*TGF*β), *Wnt*, Phosphoinositide 3-kinase (*PI3K/AKT*) and Focal adhesion kinase (*FAK*) signalling were preserved across the three modules as were remodelling and cell adhesion processes (**S1 Fig**). This analysis overview resonated with the current literature on mechanisms underlying keloid pathobiology, thus validating our data.

### An *in situ* microdissection approach leads to improved accuracy and sensitivity of differential gene expression over monolayer culture and whole tissue biopsy dissection

We compared differential gene expression of KD with NS samples from whole tissue biopsy, monolayer culture and *in situ* LCM, in order to determine both experimental approach most representative of the keloid microenvironment and also the method most likely to identify important or novel biomarkers/pathways.

**Fig 2:**
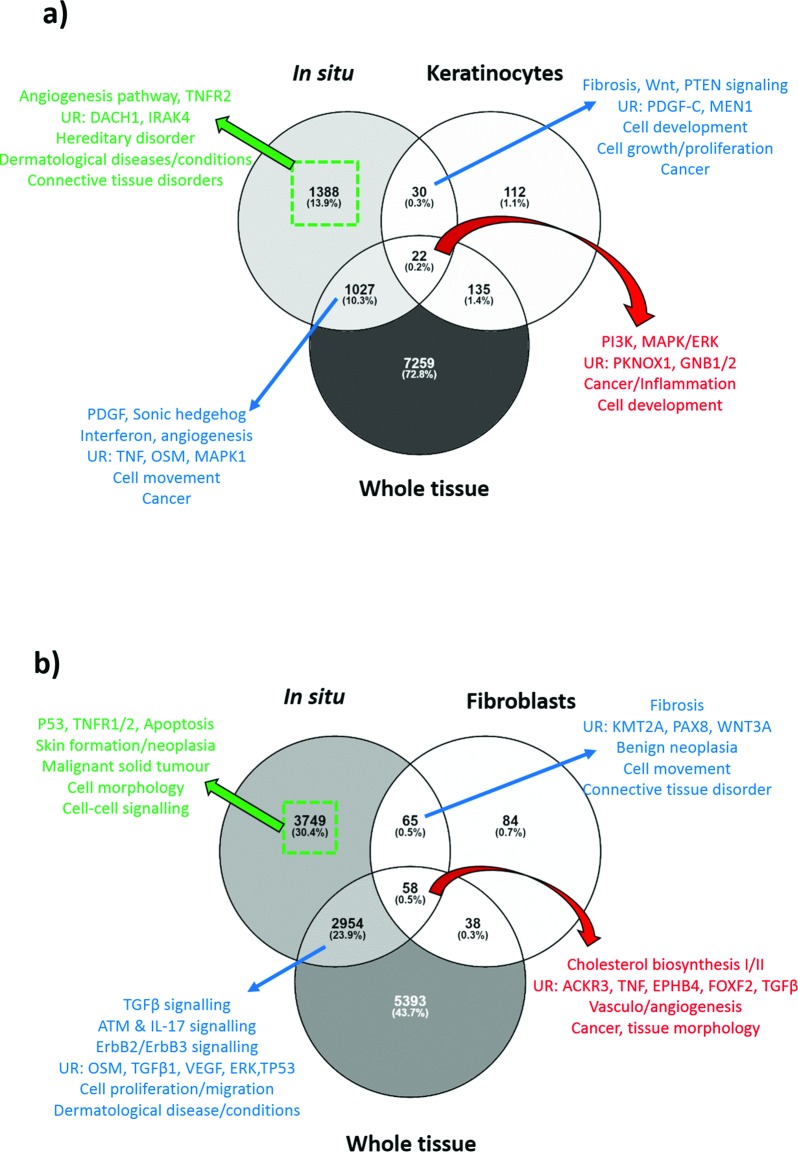
Comparison of *in situ* microdissection KD expression to whole tissue biopsy and monolayer culture expression. (A) Venn diagram comparing microdissected keloid epidermal expression to whole tissue and keratinocyte culture expression. The red arrow and text indicates where all 3 methods overlap and the results of enrichment using Ingenuity Pathway Analysis (IPA) for this group. The blue arrows and text indicate where either alternative method overlaps with *in situ* expression and the associated enrichment for that group. The green arrow and text indicates the enrichment analysis result for the 1388 genes that were identified in the microdissection group alone. (B) Venn diagram comparing micro-dissected keloid dermal expression to whole tissue and fibroblast culture expression. The red arrow and text indicates where all 3 methods overlap and the results of enrichment using IPA for this group. The blue arrows and text indicate where either alternative method overlaps with *in situ* expression and the associated enrichment for that group. The green arrow and text indicates the enrichment analysis result for the 3749 genes that were identified in the microdissection group alone. ACKR3, atypical chemokine receptor 3; ATM, ataxia telangiectasia mutated; DACH1, dachshund family transcription factor 1; EGF, epidermal growth factor; EPHB4, ephrin (EPH) receptor B4; FOXF2, forkhead box F2; GNB, guanine nucleotide binding protein (G protein); IL, interleukin; IRAK4, interleukin-1 receptor associated kinase-4; KMT2A, lysine (K)-specific methyltransferase 2A; MAPK/ERK, mitogen-activated protein kinase; MEN1, menin; OSM, oncostatin M; PAX8, paired box 8; PDGF, platelet-derived growth factor; PI3K, phosphoinositide 3-kinase; PKNOX1, PBX/knotted 1 homeobox 1; PTEN, phosphatase and tensin homolog; TGFβ, transforming growth factor beta; TNF, tumour necrosis factor; TNFR2, tumour necrosis factor receptor 2; TP53, tumour protein 53; UR, upstream regulators; VEGF, vascular endothelial growth factor.

This was achieved by first comparing the DEG produced by each approach to establish any overlap or disparity. As seen from Venn diagrams [28] (**Fig 2A and 2B**) only 0.2% and 0.5% of DEG were common to all three approaches in epidermis and dermis respectively. Given that whole tissue biopsy incorporates both epidermis and dermis, it’s clear the microdissection approach produces the highest number of DEG specific to either layer. This data was uploaded to Ingenuity Pathway Analysis (IPA) Software (Ingenuity® Systems, www.ingenuity.com), which organised the DEG into known canonical pathways, biological functions, upstream regulators and interactive networks. For both epidermis and dermis, while all three groups captured the predominant epidermal (*PI3K*, *MAPK/ERK*) and dermal (*TGF*β) expression, the microdissection approach incorporated the essential elements of both whole tissue and monolayer expression, providing a more complete picture of overall expression. Also, there were a significant number of DEG in the microdissection group alone that were not identified with monolayer or were averaged out in whole tissue analysis. This is due to the dilution effect of looking at whole tissue as transcriptomes from different cell types that are pooled together, which reduces the sensitivity.

**Fig 3:**
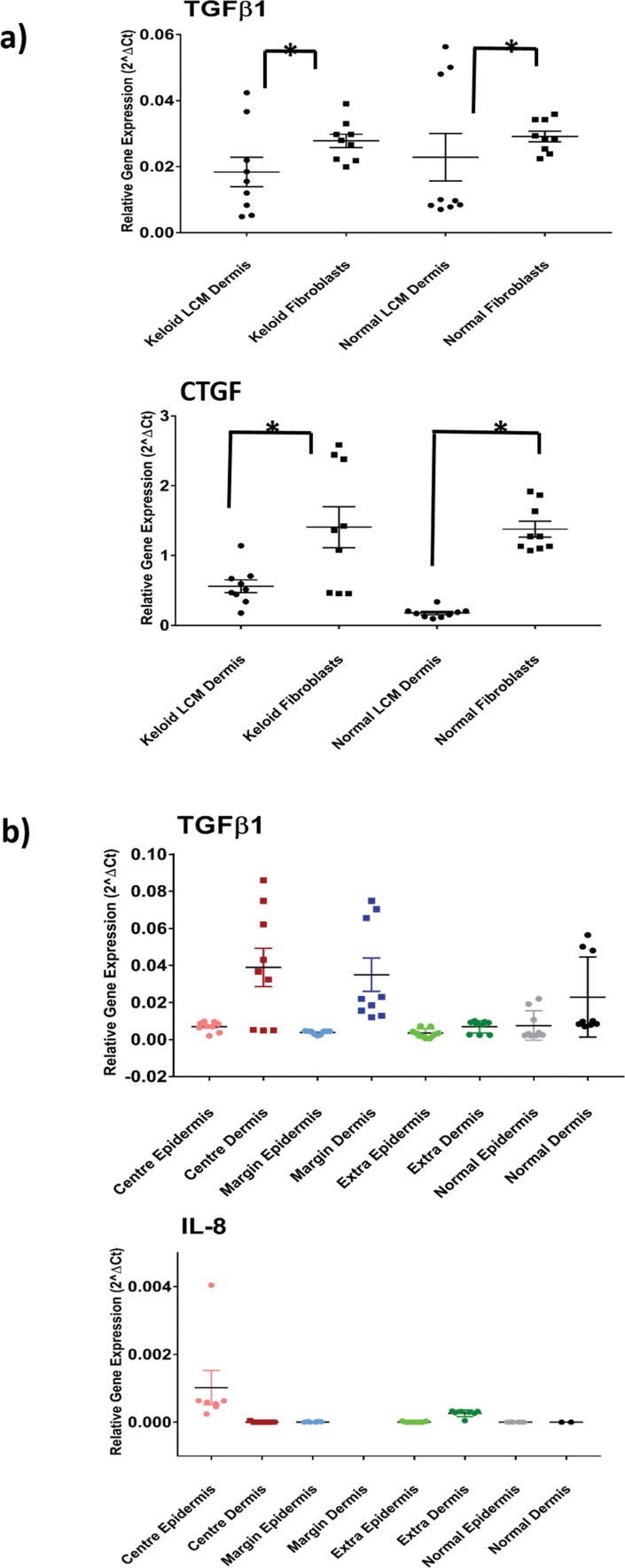
Site-specific contribution to differential gene expression in KD. (A) Comparison of gene expression between laser-captured dermal tissue (*in situ*) and fibroblasts for both keloid and normal skin. qRT-PCR graph for both *TGFβ1* and *CTGF* (additional examples found in **S2A Fig**). All data are mean ± SEM for at least three independent experiments. B) qRT-PCR for *TGFβ1* and interleukin-8 (*IL-8*) showing relative contributions of different keloid sites to overall expression and comparison with normal skin (additional genes available in **S2B Fig**). Data are mean ± SEM where * p-value <0.05 using Student’s *t* test and ANOVA with Tukey post hoc correction. CTGF, connective tissue growth factor; TGFβ, transforming growth factor beta.

In addition to comparing DEG between approaches, we examined the difference in degree of expression. For this, we compared expression between *in situ* micro-dissected dermis, captured by LCM and 2D *in vitro* cultured fibroblasts for genes known to be dysregulated in KD. Representative graphs are shown in **Fig 3A,** which demonstrate significantly increased expression of both *TGF*β*1* and *CTGF* with *in vitro* monolayer fibroblasts compared with micro-dissected tissue, for both KD and NS. This was also true for the rest of the genes analysed (**S2 Fig**). This might be somewhat expected considering 2D culture is a static physical environment, maintained with exogenous often undefined (foetal bovine serum) medium that lacks stimuli from other cell types and can alter cell morphology/polarity and phenotype [29]. These factors affect expression, with monolayer often resulting in higher-magnitude changes [12]. In this case, the overexpression seen with monolayer culture vs *in situ* microdissection approach falsely minimises the degree of gene upregulation in keloid compared with NS dermis. Given the sensitivity and accuracy of degree of expression, we concluded our *in situ* LCM approach would be the most fruitful going forward.

### *In situ* microdissection reveals keloid sites contribute disproportionately to differential gene expression

Given the advantages of LCM *in situ* tissue capture, we then sought to use this technique to define the contribution of different sites within the keloid lesion to specific gene expression as well as distinguish epidermal from dermal signalling. To achieve this, we performed qRT-PCR for a number of genes known to be dysregulated in KD (**S2 Fig**), of which two are represented in **Fig 3B.** We demonstrated that *TGF*β*1* signalling is largely attributed the keloid centre as compared to the margin or extralesional dermis and identified IL-8 upregulation as localised largely to keloid centre epidermis. By examining the keloid as separate components we were able to attribute specific expression to key sites, with the precision necessary for therapeutic targeting.

### Enrichment of the site-specific microdissected keloid epidermis suggests an active role in the pathogenesis of keloid disease

**Fig 4:**
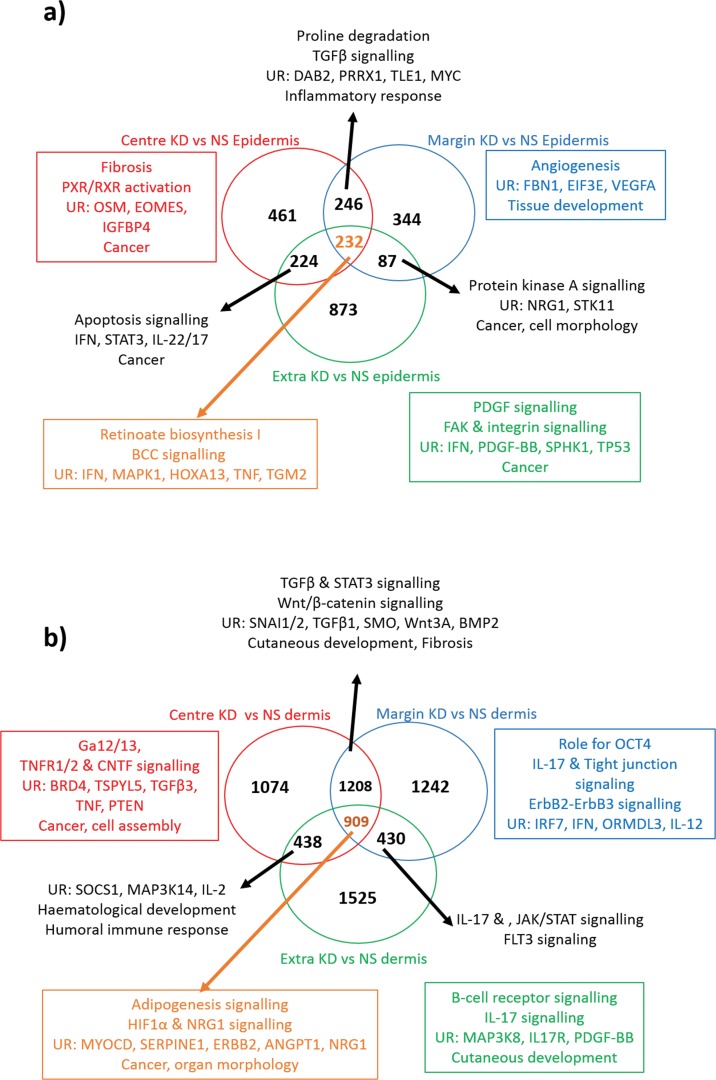
Gene enrichment analysis of microdissected site-specific keloid disease. Venn diagram of keloid disease (KD) centre, margin and extralesional expression vs normal skin (NS), where A) refers to the epidermis and B) refers to the dermis. The red circle and text refer to the centre vs NS alone, the blue to margin vs NS and the green to extralesional keloid site alone vs NS. The black arrows and text refer to the enrichment results of where the expression of the indicated sites overlap. The orange arrow and text refers to the enrichment analysis of the indicated number of genes in common to all three keloid sites over NS. In both A) and B) enrichment analysis was performed with Ingenuity Pathway Analysis (IPA) and included canonical pathways, diseases & functions, networks and upstream regulators of interest. ANGPT2, angiopoietin 2; BCO1, beta-carotene oxygenase 1; BMP2, bone morphogenetic protein 2; BRD4, bromodomain containing 4; DAB2, Dab, mitogen-responsive phosphoprotein, homolog 2 (drosophila); EGF, epidermal growth factor; EIF3E, eukaryotic translation initiation factor 3 subunit E; EOMES, eomesodermin; FBN1, fibrillin 1; FLT3, fms related tyrosine kinase 3; GSTP1, glutathione s-transferase pi 1; HIF, hypoxia-inducible factor; HOXA13, homeobox A13; IFN, interferon; IGFBP, insulin-like growth factor binding protein; IL, interleukin; IRF, interferon regulatory factor; JAK, janus kinase; MAPK, mitogen-activated protein kinase; MYC, c-Myc; MYOCD, myocardin; NRG1, neuregulin-1; OCT4, octamer-binding transcription factor 4; ORMDL3, orosomucoid like 3; OSM, oncostatin M; PDGF, platelet-derived growth factor; PTEN, phosphatase and tensin homolog; PXR, pregnane X receptor; PRRX1, paired related homeobox 1RXR, retinoid X receptor; SERPINE1(PAI-1), serpin peptidase inhibitor. Clade E (plasminogen activator inhibitor 1); SMO, smoothened; SNAI1, snail family zinc finger 1; SOCS, suppressor of cytokine signalling; SPHK, sphingosine kinase; STAT, signal transducer and activator of transcription; STK11, serine/threonine kinase 11; TGF, transforming growth factor; TGM2, transglutaminase 2; TLE1, transducin like enhancer of split 1; TNF(R), tumour necrosis factor (receptor); TP53, tumour protein 53; TSPYL5, testis-specific protein Y encoded like 5; UR, upstream regulators; VEGF, vascular endothelial growth factor.

We employed LCM to look at the epidermis as a separate entity from the dermis, which highlighted a number of DEG that have previously been overlooked with alternative methods of dissection. Generally for the epidermis, the margin and centre shared more upregulated genes than either did with the extralesional tissue, however, the centre and extralesional sites had more downregulated genes in common than either did with the margin. To interpret the expression differences and similarities between epidermal sites, the data was uploaded to IPA (**Fig 4A**). The centre keloid epidermis (KE) alone was characterised by fibrosis, inflammation and apoptosis. When taken together with the margin KE, collagen turnover and inflammation were distinguished as key events. The upstream regulators *TGF*β, PRRX1 and *DAB2* supported an EMT hypothesis. The identification of angiogenesis and cell-cell signalling networks as well as *VEGF* as an upstream regulator upheld the margin as the active site of keloid and emphasised the role of the epidermis in this process [7]. There were 232 DEG common to all three sites of the KE compared with NS epidermis (NSE). Interestingly, retinoate biosynthesis was revealed to be the top canonical pathway in this group with key molecules including *IFN*, *TNF*, *HOXA13* and *MAPK*1 proposed as upstream regulators.

### Enrichment of the site-specific micro-dissected keloid dermis supports the presence of epithelial-mesenchymal transition, immune modulation and keloid margin-related migration

With regard to the keloid dermis (Kd), there was a significant increase in shared expression between centre and margin than with extralesional tissue, for both up and downregulated genes (**Fig 4B**). Compared with NS dermis (NSD), there were 1208 DEG common to centre and margin Kd, where *TGF*β signalling was identified by IPA as a top canonical pathway, as might be expected in KD. The proposed upstream regulators in this group included *SNAI1*, SNAI2, *SMO*, *Wnt3A* and *BMP2*, which not only influence cell growth, proliferation and fibrosis but are also all involved in EMT [30–33]. When we examined all three sites of the microdissected Kd together, we identified angiogenic factors (*HIF1*α, *ANGPT2*) and migration regulators (ErbB, SERPINE1) to be in common. Enrichment of the margin Kd highlighted migration regulators (ErbB & tight junction signalling), the potential existence of embryonic stem cell markers (*OCT4*), which were previously identified in the microvessels of keloid associated lymphoid tissue [34] and immune response modulators (*IL-17*, *IL-12* & *IRF7* [35]), much of which were shared at its overlap with extralesional dermis expression. These processes are consistent with the signature expected of an invading tumour margin [36].

### The microdissected keloid epidermis expresses an activated, pro-inflammatory profile with the potential for epithelial-mesenchymal transition

In addition to expression overview and enrichment, we investigated the individual DEG of micro-dissected sites for both KE and Kd compared with NS. A full list of the top 100 upregulated and 50 downregulated genes is available for each site in **S5–S16 Figs**. Additionally, a detailed table of expanded genes for each of the symbols below can be found in **S2 Table.**

For the epidermis, the most significantly dysregulated genes were common to all three sites and indicated an activated, hyper-proliferative, pro-inflammatory and mesenchymally-poised epithelium. Keratin 6α and 6β were both significantly upregulated in all three epidermal sites, supporting the hyper-proliferation seen on keloid histology [1]. *K6* is induced in keratinocytes following injury where it is associated with migration through *Src* regulation [37, 38]. This activated epidermal expression [39] is reversible on wound closure and NSE (except the hair follicle) does not express *K6* [40]. Mucin-like 1 (*MUCL1*) was also significantly overexpressed in the micro-dissected epidermis and has been associated with aggressive breast tumorigenesis and recurrence [41]. Interestingly, it was demonstrated that *MUCL1* may be required for proliferation of *ErbB2*-overexpressing breast cells [41], which was identified as overexpressed here in margin Kd. The inflammation-associated scavenger receptor *CD36* is also a marker of epidermal activation and not present in NS keratinocytes without specific stimuli [42]. It has been shown to disappear from hypertrophic scars with age whereas keloid scars maintained their *CD36* expression [43, 44], which is supported here by *CD36* upregulation in all 3 sites of the KE. The role of *CD36* in signal transduction suggests it may contribute to epithelial-mesenchymal signalling and it has been shown to affect the secretion of *TGF*β*1* [45], which is dysregulated in KD [46, 47].

The presence of EMT in KD, is supported by our finding of *S100A8*, *WDR66* and *AKR1B10* overexpression in all 3 micro-dissected epidermal sites. The knockdown of keratinocyte *S100A8*, recently shown to be upregulated in both keloid and hypertrophic scar epidermis, resulted in a failure to activate co-cultured fibroblasts and reduction in dermal fibrosis [48]. As a mediator of neutrophil migration [49, 50], *S100A8* is found in the supra-basal epidermis following injury, but gradually returns to baseline in a normally healed wound [51]. The role of *S100A8* in conversion of wounded keratinocytes to a migratory phenotype may represent a potential link to EMT and contribution to tumorigenesis [51]. Also implicated in EMT is the protein WDR66, which in oesophageal carcinoma has been shown to affect vimentin and occludin expression where its knockdown attenuated both cell proliferation and motility [52]. While we did not find altered vimentin expression, we did identify upregulation of fibronectin (*FN1*) (p = 0.06) and α-*SMA/ACTA2* (p = 0.033) in the micro-dissected centre KE. Our data also identified a significant upregulation of *AKR1B10* (as well as *AKR1B1* and *AKR1B15*) in all three sites of the KE. This enzyme has a key role in the metabolism of retinoic acid (RA) and our recent study found the induced overexpression of *AKR1B10* in NS keratinocytes resulted in significant downregulation of E-cadherin [23]. Additionally, the treatment of keloid fibroblasts with AKR1B10-overexpressing keratinocyte medium resulted in upregulation of *TGF*β1, Collagen I and collagen III, supporting a role for pathological epithelial-mesenchymal interactions (EMI) in keloid pathogenesis.

EMI encompass the essential cross-talk that governs the epidermal-dermal relationship in the skin and in addition to a multitude of essential organ development and physiologic processes, are essential for successful wound healing. Dysregulation of the processes involved in EMI (e.g. malfunction of negative feedback loops) can lead to abnormal wound healing and fibrosis or contribute to tumorigenesis [53, 54]. Previous exploration into the contribution of these EMI to KD, have highlighted the significance of the epidermis in the formation and maintenance of this fibrotic scar [55, 56].

*BMP2*, a member of the *TGF*β superfamily, was downregulated in our microarray data and has been shown to attenuate renal fibrosis, by reversing *TGF*β1-induced EMT and cellular fibrotic markers [32]. Similarly, the loss of claudin-4 (*CLDN4*) and *CLDN23*, integral components of the tight junctions that maintain epithelial cell contacts [57] and downregulated here in the micro-dissected KE, are strongly implicated in EMT, potentially through E-cadherin modulation and thought to be negatively regulated by *TGF*β [58–60]. We also identified significant upregulation of *NOTCH4* in all 3 epidermal sites and centre and margin Kd, which is linked with cancer stem cell activity and interestingly has very recently been associated with mesenchymal-epithelial transition (MET) [61, 62].

**Fig 5:**
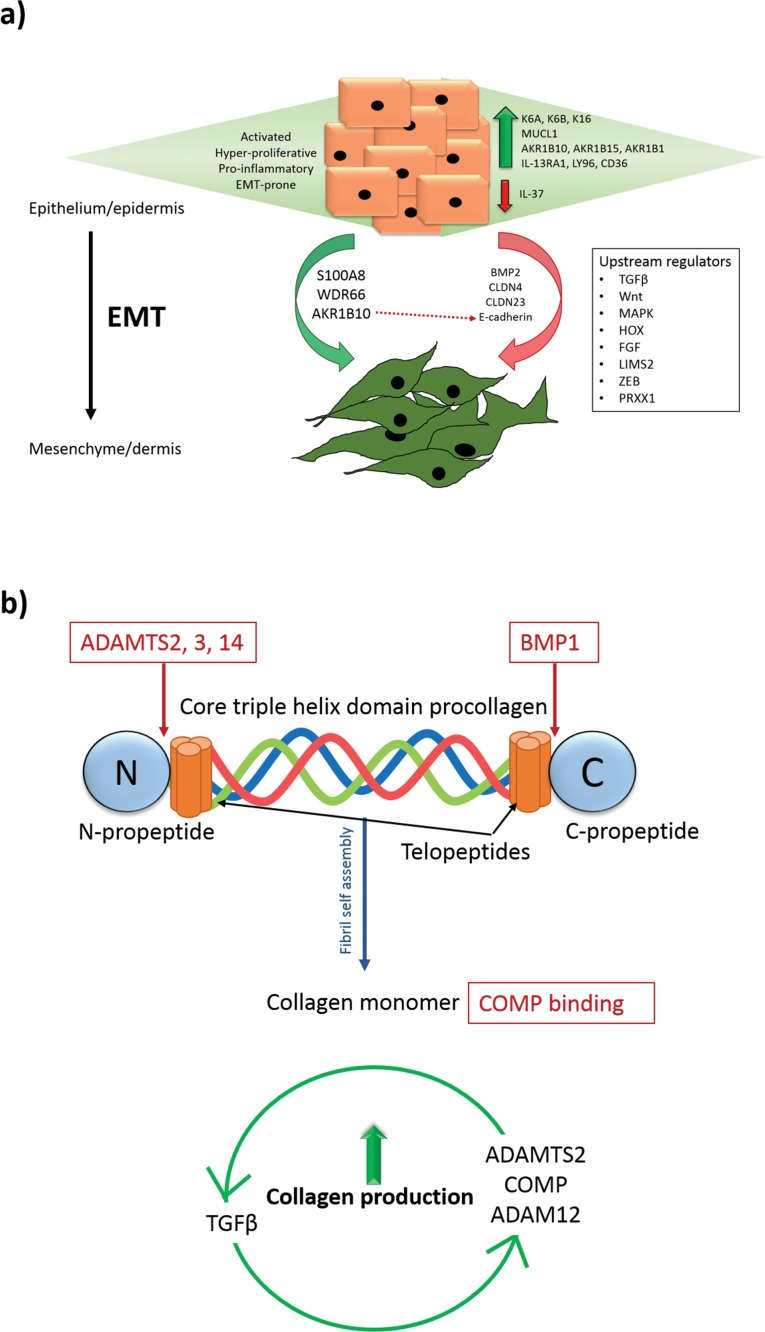
*In situ* microdissection expression contributing to epithelial-mesenchymal transition (EMT) and collagen production in KD. A) Schematic diagram of the differentially expressed genes (DEG) in KD that contribute to an activated, hyper-proliferative and inflammatory epidermis. Also depicted are DEG from our microarray data hypothesised to contribute to EMT through upregulation (green arrow) or downregulation (red arrow), which are described along with the upstream regulators identified on enrichment analysis of our microarray data and which have been previously implicated in the EMT process. B) Schematic diagram depicting where ADAMTS and BMP cleave the procollagen peptides to form tropo-collagen and allow collagen fibril assembly necessary for collagen turnover. Once a collagen monomer, it may bind COMP. Collagen production in KD may be increased by the potential existence of positive feedback loops between ADAMTS2/COMP/ADAM12 and TGFβ. ADAM, a disintegrin and metalloproteinase; ADAMTS, a disintegrin and metalloproteinase with thrombospondin motifs; AKR1B10, aldo-keto reductase family 1, member 10; BMP, bone morphogenetic protein; CLDN, claudin; COMP, cartilage oligomeric protein; FGF, fibroblast growth factor; HOX, homeotic gene subset; IL, interleukin; K, keratin; LIMS2, LIM zinc finger domain containing 2; MAPK, mitogen-activated protein kinase; MUCL1, mucin-like 1; S100A8, S100 calcium-binding protein A8; TGFβ, transforming growth factor beta; WDR66, WD repeat domain 66; ZEB, zinc finger E-box-binding proteins.

A role for EMT has previously been implicated in the pathogenesis of KD [63, 64]. EMT is a potentially reversible process whereby epithelial cells lose adhesion properties (downregulation E-cadherin, *CLDN4* and *CLDN23*) and gain migratory and invasive characteristics, transforming into mesenchymal cells (upregulation *FN1* and *ACTA2*) [65]. Here, we present DEG that in combination support a role for EMT or at least “partial EMT” in KD (**Fig 5A**).

### Microdissected keloid dermis expression reinforces the significance of TGFβ in KD pathogenesis through its regulation of the collagen network

Collagen is the major structural protein of the ECM and fibrillar collagens (type I & III) are present in abundance in KD [1]. Fibroblasts are the most common producers of collagen and are subject to complex autocrine and paracrine cytokine signals to regulate its formation, of which TGFβ is crucial [66, 67]. In combination with these cytokines there are a number of enzymes and proteins that through cleavage or binding can affect collagen turnover [68].

Within the microdissected centre and margin Kd, we identified dysregulation of a number of members of the ADAMTS family of enzymes, which are associated with tissue morphogenesis, remodelling, inflammation and angiogenesis [69]. Of these, *ADAMTS14* and *ADAMTS2* were the most significantly upregulated and are both pro-collagen N peptidases (pNP), responsible for the cleavage of type I and II pro-collagen necessary for fibril assembly and collagen biosynthesis (**Fig 5B**) [70, 71]. Interestingly, *BMP1*, responsible for the cleavage of the C-proteinase is also upregulated in centre and margin Kd (**Fig 5B**) [72]. The *ADAMTS* enzymes are associated with Dupuytren’s disease [73], craniofacial fibrosis [74], Ehlers Danlos [75] and cancer [76, 77], with *ADAMTS2*-knockout mice demonstrating skin fragility [78] and reduced liver fibrosis *in vivo [79].* More recently, studies have argued for *ADAMTS* involvement in a positive *TGF*β feedback loop, whereby *ADAMTS2* is induced by but also targets *TGF*β [80, 81]. To date the best-described inhibitor of *ADAMTS* is *TIMP3* [70, 82, 83], which we found to be significantly downregulated in our microarray data in both centre (p = 0.026) and margin (p = 0.037) Kd. Interestingly, we identified upregulation of disintegrin and metalloproteinase *ADAM12*, the member of a family closely related to the ADAMTS group of proteins and which was also suggested to be involved in a positive feedback loop with *TGF*β, resulting in continuous collagen production [84]. This proteinase is upregulated in several cancers and fibroses and was previously identified as upregulated in the keloid centre, where it was thought contribute to tissue remodelling [5, 85, 86]. Through its association with *TGF*β, *ADAM12* has been implicated in EMT [87].

Also involved in collagen fibril assembly, is cartilage oligomeric matrix protein (*COMP*), which was significantly upregulated in both centre and margin Kd. *COMP* binds with affinity to collagens, especially collagen I, and has been previously identified in KD [88], where similar to *ADAMTS* it may be involved in a positive *TGF*β feedback loop [89, 90]. Another member of the collagen matrix regulators and also previously investigated in KD is collagen triple helix repeat containing 1 (*CTHRC1*), which was demonstrated to decrease *TGF*β-induced keloid fibroblast collagen I expression [91]. However, despite the seemingly contradictory descriptions of the negative effect of *CTHRC* on collagen I expression [92, 93], it has been widely correlated with tissue invasion and migration, where its expression was induced by *TGF*β [94, 95]. Here, we found *CTHRC1* significantly upregulated in microdissected centre and margin Kd. This may represent the result of a feedback mechanism to counteract the expression of *TGF*β, *ADAMTS* and *COMP* but without further investigation the mechanism underlying *CTHRC1* overexpression in keloid scars remains to be fully elucidated. In addition to *ADAMTS*, *ADAM12*, *COMP* and *CTHRC1*, we identified asporin (*ASPN*) and Wnt1-inducible-signaling pathway protein 1 (*WISP1*) as upregulated in both centre and margin Kd, which are also correlated with *TGF*β expression. *ASPN*, a small leucine-rich proteoglycan though to regulate tumour microenvironment, is known to bind *TGF*β [96, 97] and has previously been found to be upregulated in the margin of KD [98]. The pro-proliferative *WISP1*, a member of the matricellular CCN family, was detected in Dupuytren’s disease [99] and is strongly associated with liver [100] and lung fibrosis, where it was induced by *TGF*β1 [101] and implicated in EMT.

*TGF*β is considered a master regulator in KD, involved in several positive and negative feedback loops that culminate in the net production of excess ECM through angiogenesis, proliferation, inflammation, differentiation processes and as indicated here, collagen deposition (**Fig 5B**) [102, 103]. *TGF*β is also a major player in EMT, where it effects change at transcriptional and translational levels through both Smad and non-Smad pathway signalling [104–108]. While *TGF*β*3* was significantly upregulated in the keloid centre and margin on microarray, *TGF*β1 was confirmed as upregulated in Kd compared with normal skin dermis using qRT-PCR (**Fig 3A**).

### *In situ* microdissection expression indicates the potential contribution of IL-13, IL-17 and IL-37 to the inflammatory process underlying keloid disease

The inflammatory phase of wound healing is a spatially and temporally precise process, essential to the supply of growth factor, chemokine and cytokine signalling necessary for repair. However, prolonged inflammation can result in impaired wound healing, leading to chronic wounds or excess scarring [109, 110]. KD is associated with an exaggerated inflammatory response [6, 111]. Prolongation of the inflammatory phase with extended residency of these factors promotes proliferation, angiogenesis and increased deposition of ECM [112]. Interleukins are a group of secreted cytokines central to the inflammatory process that have an incompletely understood role in KD and may represent potential therapeutic targets.

**Fig 6:**
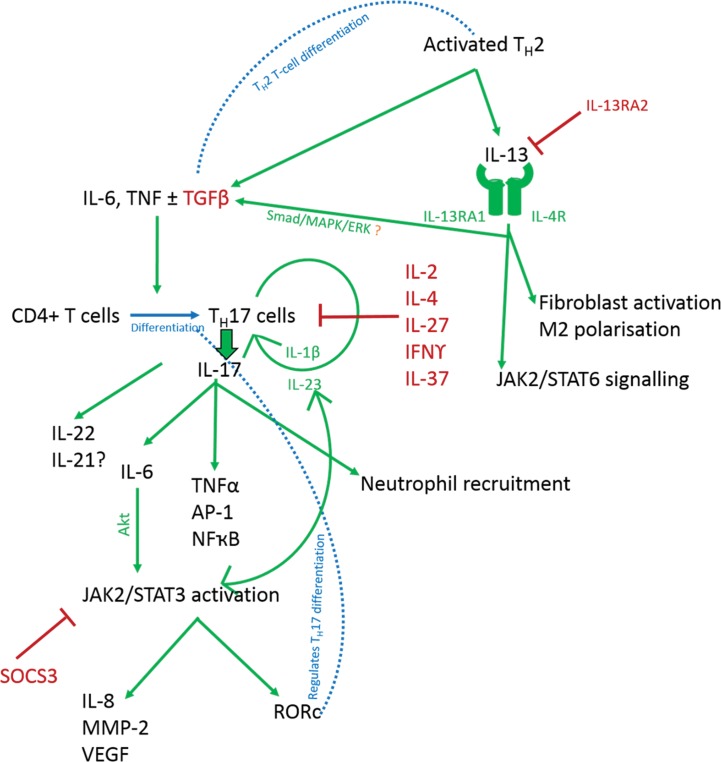
Cytokine relationship with potential inflammatory effects in KD. Schematic diagram of the possible relationships existing between a number of cytokines and growth factors identified as dysregulated in KD microdissected epidermis and dermis in our microarray data. This figure should be correlated with Table 2 where the direction and fold change for each of these molecules can be found for each site within keloid epidermis and dermis. AP-1, activating protein 1; IL, interleukin; INF, interferon; JAK, janus kinase; MMP, matrix metalloproteinase; NFκB, nuclear factor kappa B; ROR, retinoic acid-related orphan receptor c; STAT, signal transducer and activator of transcription; TGFβ, transforming growth factor beta; TNF, tumour necrosis factor.

*IL-13*, a potent fibrosis-promoting cytokine secreted by activated T_H_2 T-cells [113, 114], has been shown to increase collagen I & III production in keloid fibroblasts *in vitro* [115]. We found significant upregulation of *IL13RA1* in KE, epidermal and dermal upregulation of IL-4R and downregulation of *IL13RA2* in the microdissected Kd (**Table 2**). Together, *IL-13RA1* and *IL-4R* bind both *IL-13* and *IL-4* with high affinity to activate *JAK/STAT6* signalling (**Fig 6**) [116]. *IL-13RA2* is largely considered a high affinity decoy receptor thought to inhibit *IL-13* signalling *in vivo* and protect against fibrosis [117, 118]. Although this has been disputed [116], *IL-13* inhibition attenuated fibrosis and *IL-13RA2*-knockout mice have demonstrated enhanced *IL-13*-mediated responses *in vivo* [119–121]. Our current microarray findings, combined with previous evidence of KD containing an inflammatory niche populated by M2 macrophages [6], known to be *IL-13* recruited, supports an overexpression of *IL-13* in KD.

**Table 2:** Dysregulation of cytokines in site-specific KD microarray relating to IL-13, IL-37 and IL-17. All have p-value < 0.05. See Fig 6 for relationships.

Molecule	Keloid site	Centre: direction & fold change	Margin: direction & fold change	Extralesional: direction & fold change
**IL-13RA1**	*Epidermis*	↑ 8.45	↑ 4.8	↑ 3
**IL-13RA2**	*Dermis*	↓ 10.8	↓ 8.67	↓ 9.66
**IL-37**	*Epidermis*	↓ 6.51	↓ 4.85	↓ 10.66
**IL-17RA**	*Epidermis*	-	-	↓ 2.6
**IL-17RA**	*Dermis*	↓ 2.9	↓ 2.35	↑ 3.36
**IL-1β**	*Dermis*	-	↑ 3.83	↑ 4.61
**IL-23A**	*Dermis*	-	-	↑ 2.73
**IL-6R**	*Epidermis*	-	↑ 4.06	-
**IL-21R**	*Dermis*	-	↑ 2.64	↑ 2.01
**IL-2RA**	*Dermis*	-	↑ 2.95	-
**IL-4R**	*Epidermis*	↑ 2.31	-	-
**IL-4R**	*Dermis*	↑ 5.97	↑ 11.41	↓ 4.65
**IL-27**	*Dermis*	↑ 2.34	↑ 1.84	↑ 2.15
**STAT3**	*Dermis*	-	↓ 2.02	↓ 2.1
**SOCS3**	*Epidermis*	-	-	↑ 3.64
**SOCS3**	*Dermis*	↑ 4.32	↑ 5.68	↑ 5.69
**IL-8**	*Dermis*	-	-	↑ 12.27
**RORc**	*Dermis*	↓ 4.92	-	-

IL, interleukin; R, receptor; RA, receptor alpha; A, alpha; STAT, signal transducer and activator of transcription; SOCS, suppressor of cytokine signalling; ROR, retinoid-related orphan receptor.

In addition to *IL-13* dysregulation, in all 3 KE sites compared with NS epidermis we identified loss of *IL-37*, a relatively new member of the interleukin-1 (*IL-1*) family, which described as anti-inflammatory has been shown to decrease the expression of *IL-6*, *IL-1*β, *TNF*α and *IL-17* [122], all of which are associated with KD [13, 112]. It is thought *IL-37* is involved in a negative feedback loop to control excess inflammation, whereby *IL-37* induction by *TNF*α or toll-like receptors (TLR) results in suppression of *TNF*α and inhibition of pro-inflammatory cytokine release (**Fig 6**) [123]. While *IL-37* itself has not previously been investigated in KD, this finding is supported by altered expression of *IL-17*, *IL-1*β and *TNF*α in our microarray data.

IL-17 signalling was dysregulated in common to both margin and extralesional Kd sites on enrichment analysis (**Fig 4B**). The pro-inflammatory *IL-17* is produced by a subset of activated CD4+ T-cells, namely T_H_17 and a subset of innate lymphoid cells termed ILC3s, whose differentiation and cytokine production is regulated by a complex interplay of molecules [124–126]. Studies have shown that *IL-6* and *TGF*β initiate T_H_17 differentiation, *IL-23* in an autoregulatory feedback loop with *IL-1*β is responsible for the maintenance of *IL-17* and that *IL-2*, *IL-27*, *IL-4* and *IFN*ϒ are negative regulators [124, 127]. While we know KD shows an increased T-cell infiltrate in the dermis and that there is an inflammatory niche driven by the *IL-17/IL-6* axis, the complex interplay of this signalling mechanism remains incompletely understood [6, 13, 128]. Here, we identify an imbalance in inflammatory cytokine signalling, which alters between the three KE and Kd sites compares to NS (**Table 2**). Within the literature the association between these cytokines and their regulators/substrates is described with some variability and **Fig 6**depicts an interpretation of these relationships and how they may interact in KD. The *IL-17* environment is both cell-type and context-type dependent in contribution to neutrophil recruitment, angiogenesis and invasion [129, 130]. IL-17 is known to be involved in other fibrotic conditions [131–133] and in KD it may be that *IL-17* expression differs between different sites within the keloid scar and that ongoing paracrine signalling produces the dynamic expression seen here.

The dysregulation of *IL13*, *IL-37* and *IL-17* in KD from our microarray data are likely interconnected (**Table 2**) and the mechanisms underlying modulation of KD by these interleukins requires further elucidation to determine their contribution to its pathogenesis and potential for therapeutic exploitation (**Fig 6).**

### *In situ* microdissection analysis identifies loss of tumour suppression genes that combined with an expression profile promoting therapeutic resistance may account for currently ineffective keloid management

The failure to switch on essential genes responsible for the attenuation of processes central to fibrosis can lead to exponential growth. The loss of expression of these genes can be as significant in the pathogenesis of KD as the overexpression of others. In this study, we identified a number of DEG previously associated with tumour suppression and drug resistance but not fully explored in KD.

Looking at the dermis, both *CEACAM1* and *SOX9* were found to be downregulated in keloid centre and margin compared with NSD. *CEACAM1*, a glycoprotein that mediates cell adhesion and immunity, is dysregulated in a number of cancers and considered a tumour suppressor gene [134–136]. Loss of *CEACAM1* has been implicated in the switch from superficial to pro-angiogenic, invasive tumour [137]. The concomitant downregulation of *SOX9* is likely to be linked given the correlation to *CEACAM1* in the literature to date [138–140].

We identified downregulation of *ATF3* in both centre and margin KE compared with microdissected NSE. Although not previously investigated in KD, this CREB family protein is downregulated in a number of cancers [141] and considered an adaptive-response gene with tumour-suppressing effects that to date, demonstrate dual-role cell-type dependency [142, 143]. *ATF3* promotion of apoptosis, a key process in prevention of growth and invasion, may result from *KLF6* induction of *ATF3* [144] or through its activation of *p53* [145]. We found a significant downregulation of *KLF6* in the centre KE (p = 0.02) and interestingly, *ATF3* has been shown to mediate apoptosis by anti-cancer therapies [146–148]. Also in the microdissected KE, *UGT3A2*, a member of the UDP-glycosyltransferase superfamily that plays a role in drug metabolism and which may affect detoxification of therapeutic drugs, was found to be strongly under-expressed [149, 150].

Our recent publication on the upregulation of *AKR1B10* in KE, where we hypothesised its ability to catalyse the reduction of carbonyls and xenobiotics may render keloid susceptible to chemotherapeutic resistance and thus explain some of the difficulties associated with management of KD to date [23, 151]. Both *ALDH1A1* and the aforementioned upregulation of *NOTCH4* in KE are also associated with drug resistance [152, 153]. The upregulation of these molecules and multi-drug resistant nature of keloid scars may indicate the presence of a cancer stem cell-like population within the scar, which has been touched on but not fully explored in the literature [154, 155]. Tubulin β3, class III (*TUBB3*), a cytoskeletal microtubule protein previously identified in solid tumours and extraocular fibrosis [156, 157], was also significantly upregulated in both centre and margin microdissected KE and Kd. This molecule has been linked to both overexpression of *ErbB2* [158, 159], which we found upregulated in margin Kd (p = 0.0031) and loss of *PTEN* [160, 161], which was also significantly downregulated in the margin Kd in our microarray data (p = 2.86 x 10^−5^). *TUBB3* is associated with aggressive tumorigenesis in hypoxic environments [162], where it has been linked with chemoresistance, particularly taxanes [163–165]. This may be relevant to KD where there is evidence of a similarly hypoxic environment [63, 166, 167].

KD is notoriously difficult to manage in the clinical setting, with several available treatments but no one absolutely effective therapy [168–170]. Drug resistance has formed a major part of this failure [171]. In identifying DEG that may contribute to this seemingly multi-drug-resistant disease, it may be possible to tailor management by targeting these molecules with adjuvant therapies.

### Microarray data was validated through qRT-PCR of interesting targets

**Fig 7:**
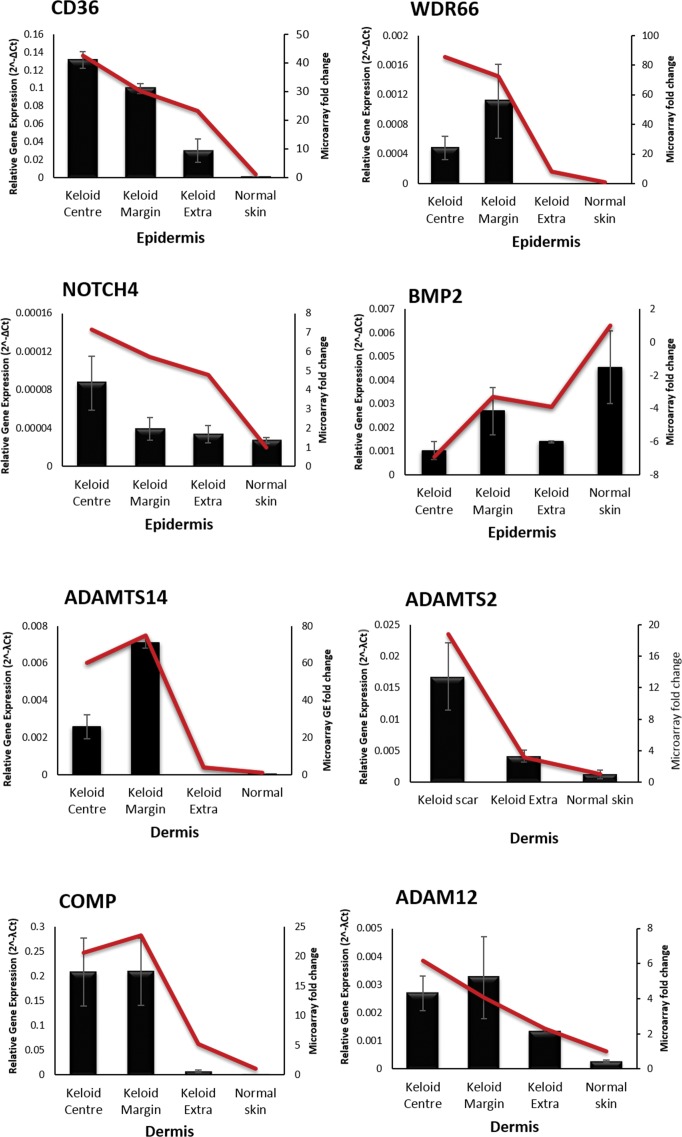
qRT-PCR validation of candidate genes. Four candidate genes were chosen from each of the epidermis and dermis for validation by qRT-PCR. The bar graphs represent the qRT-PCR data for the microdissected keloid sites and normal skin and the line graph represents the associated microarray fold change in gene expression. In all cases the line graph follows the trend of the bar graph indicating the PCR reflects the microarray, thus validating the data. Data are presented as mean ± SEM and are from at least three independent experiments. For some of the genes there was no expression in normal skin and therefore for those genes no fold change for the qRT-PCR could be generated. In the interest of standardisation of all of the graphs they were then presented with the two axes. ADAM, a disintegrin and metalloproteinase; ADAMTS, a disintegrin and metalloproteinase with thrombospondin motifs; BMP2, bone morphogenetic protein 2; CD36, cluster of differentiation 36; COMP, cartilage oligomeric protein; NOTCH4, notch 4; WDR66, WD repeat domain 66.

We chose four candidate genes from each of the KE and Kd for qRT-PCR validation of the microarray findings. For the epidermis, the dysregulation of *AKR1B10* and associated *AKR1B1*, *AKR1B15* and *ALDH1A1* all related to the retinoic acid pathway and as such were previously validated [23]. Also in the KE, we wanted to validate genes representing different areas of interest including epidermal activation and inflammation (*CD36*), EMT (*WDR66* and *BMP2*) and the possible existence of a cancer-like stem cell population (*NOTCH4*). As the most abundant protein in keloid ECM, collagen has long been investigated as a potential therapeutic target. Our identification of *ADAMTS14*, *ADAMTS2*, *COMP* and *ADAM12* represent significant alternative targets to *TGF*β and as such were chosen for validation in the dermis. qRT-PCR for these genes not only reflected the microarray findings but also preserved the site-specific differences in expression, thus validating our data (**Fig 7).**

## Conclusions and perspectives

In this study, we have combined LCM and microarray to examine KD by looking at the lesion as separate components; epidermis and dermis as well as centre, margin and extralesional sites. First, we showed this *in situ* microdissection approach was both more accurate and more sensitive than either whole tissue biopsy or monolayer cell culture methods in the dissection of the heterogeneous lesion that is keloid scar.

Through this strategy, we have distinguished several genes that are either novel or supportive of emerging literature with respect to KD pathobiology. In this study, expression patterns indicate the possible residence of a cancer-like stem cell population in KD, an area that is surprisingly under-researched in this field given the association with both EMT and drug resistance [171, 172]. The plausible presence of such a cell population in KD, which to date has been associated with an inflammatory infiltrate [13, 34, 128], provides a reasonable explanation for the persistent growth, recurrence and multi-drug resistance that are characteristic of this disorder. The LCM strategy detailed in this study could benefit the isolation and characterisation of these cancer-like stem cells from within the keloid tissue and therefore constitutes an interesting focus for future work.

The multi-level ECM regulators, *ADAMTS14* and *ADAMTS2*, make attractive KD therapeutic targets. The potential redundancy of both these proteins with *ADAMTS3*, which we did not find to be upregulated in KD, indicates possible attenuation of their effect without the consequences of complete abrogation. *IL*-*37* overexpression *in vivo* in transgenic mice has resulted in dampened *IL-6*, *IL-1*β and *IL-17* [173], which are all previously shown to be upregulated in KD [13]. This suggests therapeutic induction of IL-37 expression in KE, in order to dampen the pathologic inflammatory response, may be a prospective management strategy. Interestingly, there is differential gene expression between extralesional keloid and NS tissue. This may represent a field cancerisation effect whereby keloid tumour invasive growth is mediated by paracrine signalling with the adjacent NS and that within this extralesional perimeter the risk of keloid recurrence following treatment is greatest. Therefore, establishment of the extent of this extralesional expression divergence from that of NS may have clinical implications for future management of KD.

**Fig 8:**
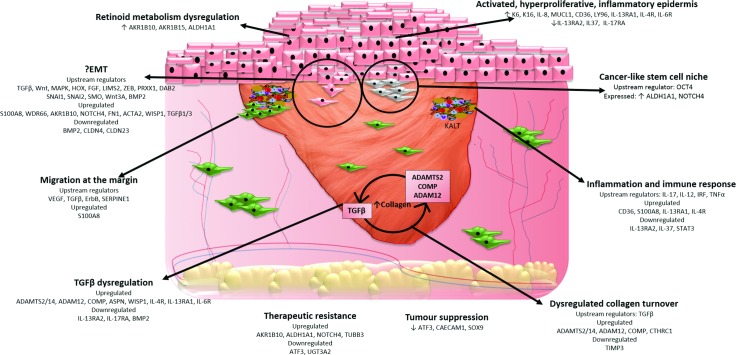
Summary figure of proposed processes and mechanisms contributing to keloid disease based on identification of DEG and subsequent analysis.

Given the clinically distinct keloid phenotypes and the morphological heterogeneity within the keloid scar, which is fully reflected here by the isolated gene expression profile of defined KD-associated lesion compartments, it is most likely a gene signature rather than a single biomarker that will prove valuable as a diagnostic tool when distinguishing KD from other cutaneous fibroses. Improved differential diagnosis prevents the morbidity and mortality associated with inappropriate management of clinically comparable conditions, some of which may have more serious consequences if improperly treated (for example; dermatofibrosarcoma protuberans and systemic sclerosis). The heterogeneity of KD has been addressed through the innovative microdissection gene expression profiling approach in this study, which has provided a better-defined gene signature of distinct regions of keloid tissue pathobiology (**Fig 8**).

It is likely that the complex nature of KD results from an interplay of simultaneously occurring processes, such that injury to the epidermis results in inflammation which then through autocrine and paracrine signalling recruits factors that trigger EMT, which in turn can trigger cellular reversion to a stem cell-like state and thus exacerbate drug resistance and recurrence [174]. While *TGF*β is an attractive therapeutic target, the pleiotropic nature of this molecule makes a simplistic TGFβ-neutralisation strategy imprudent. Therefore, it would be useful if alternative molecular mechanisms that are important in KD pathobiology but more specific to it, could be selectively targeted therapeutically. Several novel, potentially important molecular targets and KD pathobiology candidate mechanisms have been dissected here that invite and facilitate further studies. To this end, the use of LCM in this study is in keeping with previous research that highlighted the superiority of this technique with regard to identification of otherwise overlooked genes using traditional methods as well as an increased number of genes reaching significance [175–178]. Our approach therefore offers a competitive alternative to established methods of experimentation and may be of potential benefit not only to KD but also other heterogeneous conditions.

## Supporting information

S1 Table Demographic data for the samples used in this study. (DOCX)

S2 Table Expanded names for each of the gene symbols used throughout the manuscript text and figures. (DOCX)

S1 Fig Correlated modules based on assessment of eigengene plots. (DOCX)

S2 Fig qRT-PCR comparing LCM and monolayer and defining site-specific contribution to gene expression. (DOCX)

S3 Fig Top upregulated genes in keloid vs normal skin epidermis. (DOCX)

S4 Fig Top downregulated genes in keloid vs normal skin epidermis. (DOCX)

S5 Fig Top 100 upregulated genes in keloid centre vs normal skin epidermis. (DOCX)

S6 Fig Top 50 downregulated genes in keloid centre vs normal skin epidermis. (DOCX)

S7 Fig Top 100 upregulated genes in keloid margin vs normal skin epidermis. (DOCX)

S8 Fig Top 50 downregulated genes in keloid margin vs normal skin epidermis. (DOCX)

S9 Fig Top 100 upregulated genes in keloid extralesional vs normal skin epidermis. (DOCX)

S10 Fig Top 50 downregulated genes in keloid extralesional vs normal skin epidermis. (DOCX)

S11 Fig Top 100 upregulated genes in keloid centre vs normal skin dermis. (DOCX)

S12 Fig Top 50 downregulated genes in keloid centre vs normal skin dermis. (DOCX)

S13 Fig Top 100 upregulated genes in keloid margin vs normal skin dermis. (DOCX)

S14 Fig Top 50 downregulated genes in keloid margin vs normal skin dermis. (DOCX)

S15 Fig Top 100 upregulated genes in keloid extralesional vs normal skin dermis. (DOCX)

S16 Fig Top 50 downregulated genes in keloid extralesional vs normal skin dermis. (DOCX)

## Abbreviations

DEG: differentially expressed genes
ECM: extracellular matrix
EMI: epithelial-mesenchymal interactions
EMT: epithelial-mesenchymal transition
IL: interleukin
KD: keloid
Kd: keloid dermis
KE: keloid epidermis
LCM: laser capture microdissection
MET: mesenchymal-epithelial transition
NS: normal skin; disease
TGF: transforming growth factor
UV: ultraviolet
2D: 2-dimensional
