# Supplementary material for: Site-specific gene expression profiling as a novel strategy for unravelling keloid disease pathobiology
Source: PLoS One. 2017 Mar 3;12(3):e0172955. doi: 10.1371/journal.pone.0172955 (PMC5336271; doi:10.1371/journal.pone.0172955)
Supplement: S1 Table — (DOCX) [file pone.0172955.s001.docx]

**S1 Table**

**Table S1 –** Demographic data for the samples used in this study.

| **Sample No.** | **Tissue type** | **Gender, Age** | **Ethnicity** | **Time present** | **Previous Treatment** | **Location of keloid** |
| --- | --- | --- | --- | --- | --- | --- |
| **1** | **Keloid** | **F, 42yrs** | **Jewish** | **7-8 yrs** | **Nil** | **Left shoulder** |
| **2** | **Keloid** | **F, 25 yrs** | **Black/East African** | **18 mnths** | **Nil** | **Right shoulder** |
| **3** | **Keloid** | **F, 19 yrs** | **Caucasian** | **2-3 yrs** | **Nil** | **Left ear helix** |
| **4** | **Keloid** | **F, 30 yrs** | **Caucasian** | **8 yrs** | **Nil** | **Central sternum** |
| **5** | **Keloid** | **F, 32 yrs** | **Caucasian** | **13 yrs** | **Radiation, steroid, silicone** | **Breasts bilaterally** |
| **6** | **Keloid** | **F, 41 yrs** | **Jewish** | **6 yrs** | **Steroid, silicone** | **Left deltoid** |
| **7** | **Keloid** | **F, 18yrs** | **Jamaican Afrocarribean** | **1 yr** | **none** | **Bilateral ear lobes** |
| **8** | **Keloid** | **M, 23yrs** | **Jamaican Afrocarribean** | **4 yrs** | **none** | **Bilateral ear lobes** |
| **9** | **Keloid** | **F, 27yrs** | **Jamaican Afrocarribean** | **2 yrs** | **Surgery** | **Right ear lobe** |
| **10** | **Keloid** | **M, 20yrs** | **Jamaican Afrocarribean** | **2 yrs** | **Surgery** | **Chin** |
| **11** | **Keloid** | **F, 19yrs** | **Jamaican Afrocarribean** | **2 yrs** | **Surgery** | **Sternum** |
| **12** | **Keloid** | **F, 41yrs** | **Jamaican Afrocarribean** | **6 yrs** | **None** | **Sternum** |
| **13** | **Keloid** | **M, 20yrs** | **Jamaican Afrocarribean** | **15 yrs** | **None** | **Right ear lobe** |
| **14** | **Keloid** | **F, 22yrs** | **Jamaican Afrocarribean** | **3 yrs** | **None** | **Bilateral ear lobes** |
| **15** | **Normal skin** | **F, 57yrs** | **Caucasian** | **-** | **-** | **Facelift & blepharoplasty** |
| **16** | **Normal skin** | **M, 47yrs** | **Caucasian** | **-** | **-** | **Abdominoplasty** |
| **17** | **Normal skin** | **F, 19yrs** | **Caucasian** | **-** | **-** | **Bilateral breast reduction** |
| **18** | **Normal skin** | **F, 26yrs** | **Caucasian** | **-** | **-** | **Abdominoplasty** |
| **19** | **Normal skin** | **F, 41yrs** | **Caucasian** | **-** | **-** | **Abdominoplasty** |
| **20** | **Normal skin** | **F, 43yrs** | **Caucasian** | **-** | **-** | **Bilateral breast reduction** |
| **21** | **Normal skin** | **F, 43yrs** | **Caucasian** | **-** | **-** | **Bilateral breast reduction** |
| **22** | **Normal skin** | **F, 34yrs** | **Caucasian** | **-** | **-** | **Abdominoplasty** |
| **23** | **Normal skin** | **F, 47yrs** | **Caucasian** | **-** | **-** | **Mastopexy** |
| **24** | **Normal skin** | **F, 20yrs** | **Caucasian** | **-** | **-** | **Bilateral breast reduction** |
| **25** | **Normal skin** | **F, 42yrs** | **Caucasian** | **-** | **-** | **Bilateral breast reduction** |
| **26** | **Normal skin** | **F, 56yrs** | **Caucasian** | **-** | **-** | **Abdominoplasty** |
| **27** | **Normal skin** | **F, 51yrs** | **Caucasian** | **-** | **-** | **Abdominoplasty** |
