# Supplementary material for: Site-specific gene expression profiling as a novel strategy for unravelling keloid disease pathobiology
Source: PLoS One. 2017 Mar 3;12(3):e0172955. doi: 10.1371/journal.pone.0172955 (PMC5336271; doi:10.1371/journal.pone.0172955)
Supplement: S2 Table — (DOCX) [file pone.0172955.s002.docx]

**S2 Table**

**Table S2** – Expanded names for each of the gene symbols used throughout the manuscript text and figures.

| Gene symbol | Expansion |
| --- | --- |
| ACKR3 | atypical chemokine receptor 3 |
| ADAM | a disintegrin and metalloproteinase |
| ADAMTS | a disintegrin and metalloproteinase with thrombospondin motifs |
| AKR1B | aldo-keto reductase family 1, member |
| ALDH1A1 | aldehyde dehydrogenase 1 family member A1 |
| ANGPT | angiopoietin |
| AP-1 | activating protein 1 |
| ASPN | asporin |
| ATF3 | activating transcription factor 3 |
| ATM | ataxia telangiectasia mutated |
| BMP2 | bone morphogenetic protein 2 |
| BRD4 | bromodomain containing 4 |
| CCN | Cyr61, CTGF, Nov (family of matricellular proteins) |
| CD36 | cluster of differentiation 36 |
| CEACAM | carcinoembryonic antigen-related cell adhesion molecule |
| CLDN | claudin |
| CNTF | ciliary neurotrophic factor |
| COMP | cartilage oligomeric protein |
| CREB | cAMP response element binding |
| CTGF | and connective tissue growth factor |
| CTHRC1 | collagen triple helix repeat containing 1 |
| DAB2 | Dab, mitogen-responsive phosphoprotein, homolog 2 (drosophila) |
| DACH1 | dachshund family transcription factor 1 |
| EIF3E | eukaryotic translation initiation factor 3 subunit E |
| EOMES | eomesodermin |
| EPHB4 | ephrin (EPH) receptor B4 |
| ErbB | epidermal growth factor receptor family |
| FBN1 | fibrillin 1 |
| FLT3 | fms related tyrosine kinase 3 |
| FN1 | fibronectin 1 |
| FOXF2 | forkhead box F2 |
| GNB1 | G protein subunit beta 1 |
| HIF-1α | hypoxia-inducible factor-1 alpha |
| HOXA13 | homeobox A13 |
| IFN | interferon |
| IGFBP4 | insulin-like growth factor binding protein 4 |
| IL | interleukin |
| ILC | innate lymphoid cell |
| IRAK4 | interleukin 1 receptor associated kinase 4 |
| IRF7 | interferon regulatory factor 7 |
| JAK | janus kinase |
| K | keratin |
| KLF6 | kruppel-like factor |
| KMT2A | lysine methyltransferase 2A |
| LIMS2 | LIM zinc finger domain containing 2 |
| MAPK/ERK | mitogen-activated protein kinase |
| MEN1 | menin1 |
| MMP | matrix metalloproteinase |
| MUCL1 | mucin-like 1 |
| MYC | c-Myc |
| MYOCD | myocardin |
| NF-ҡB | nuclear factor kappa B |
| NOTCH | notch |
| NRG1 | neuregulin-1 |
| OCT4 | octamer-binding transcription factor 4 |
| ORMDL3 | orosomucoid like 3 |
| OSM | oncostatin M |
| PAX8 | paired box 8 |
| PDGF | platelet-derived growth factor |
| PI3K | phosphoinositide 3-kinase |
| PKNOX1 | PBX/knotted 1 homeobox 1 |
| PRRX1 | paired related homeobox 1 |
| PTEN | phosphatase and tensin homolog |
| PXR | pregnane X receptor |
| RORc | retinoic acid-related orphan receptor c |
| RXR | retinoid X receptor |
| S100A8 | S100 calcium-binding protein A8 |
| SERPINE1/PAI-1 | serpin peptidase inhibitor. Clade E (plasminogen activator inhibitor 1) |
| SMO | smoothened |
| SNAI1 | snail family zinc finger 1 |
| SNAI2 | snail family transcriptional repressor 2 |
| SOCS | suppressor of cytokine signalling |
| SOX9 | SRY (sex-determining region Y)-box 9 |
| SPHK1 | sphingosine kinase 1 |
| STAT | signal transducer and activator of transcription |
| TGFβ | transforming growth factor beta |
| TGM2 | transglutaminase 2 |
| TIMP3 | tissue inhibitor of metalloproteinases-3 |
| TLE1 | transducin like enhancer of split 1 |
| TNF | tumour necrosis factor |
| TNFR | tumour necrosis factor receptor |
| TP53 | tumor protein p53 |
| TSPYL5 | testis-specific protein Y encoded like 5 |
| TUBB3 | Tubulin β3, class III |
| UGT3A2 | UDP glycosyltransferase family 3 member A2 |
| VEGF | vascular endothelial growth factor |
| WDR66 | WD repeat domain 66 |
| WISP1 | Wnt 1 inducible signaling pathway protein 1 |
| Wnt | wingless-type MMTV integration site |
| ZEB | zinc finger E-box-binding proteins |
| α-SMA/ACTA2 | alpha smooth muscle actin/actin, alpha 2, smooth muscle, aorta |
