# Supplementary material for: Site-specific gene expression profiling as a novel strategy for unravelling keloid disease pathobiology
Source: PLoS One. 2017 Mar 3;12(3):e0172955. doi: 10.1371/journal.pone.0172955 (PMC5336271; doi:10.1371/journal.pone.0172955)

**S1 Fig**

**Figure S1** – Modules based on assessment of eigengene plots, two of which were highly correlated. A MetaCore output of these modules show that the black module enriches for TGFβ and WNT signaling, EMT and cell adhesion, cytoskeleton and tight junctions. The green module for TGFβ and Akt/PI3K signalling as well as FAK and cytoskeleton filaments.


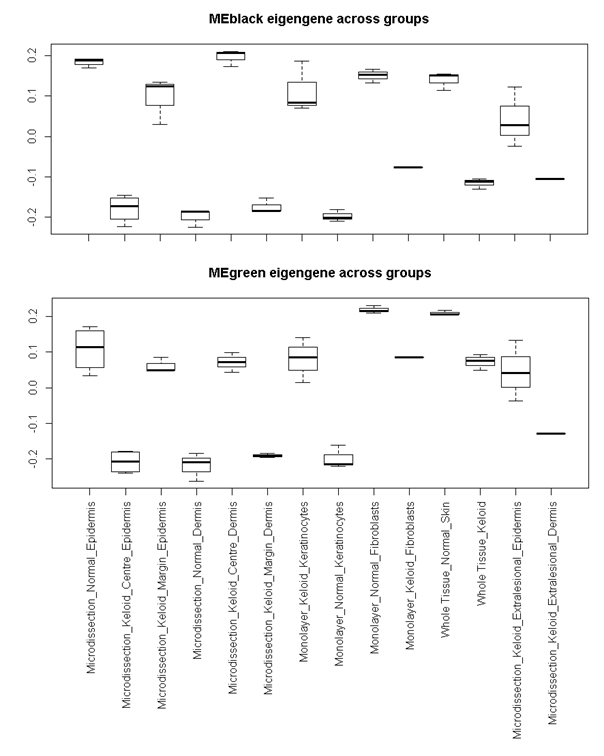

Supplement: S1 Fig — (DOCX) [file pone.0172955.s003.docx]
