# Supplementary material for: Site-specific gene expression profiling as a novel strategy for unravelling keloid disease pathobiology
Source: PLoS One. 2017 Mar 3;12(3):e0172955. doi: 10.1371/journal.pone.0172955 (PMC5336271; doi:10.1371/journal.pone.0172955)

**S2 Fig**

**Figure S2** – A) – qRT-PCR graphs showing differences in the degree of expression between LCM keloid and NS dermis and monolayer keloid and NS fibroblasts. FGF7, fibroblast growth factor 7; PDGFRA, platelet derived growth factor alpha. B) qRT-PCR graphs showing the *in situ* (LCM) contribution of both epidermis and dermis from keloid centre, margin and extralesional sites as well as normal skin to the overall gene expression of fibrosis-associated genes known to be dysregulated in KD. CTGF, connective tissue growth factor; FGF7, fibroblast growth factor 7; PDGFRA, platelet derived growth factor receptor A.

**A**


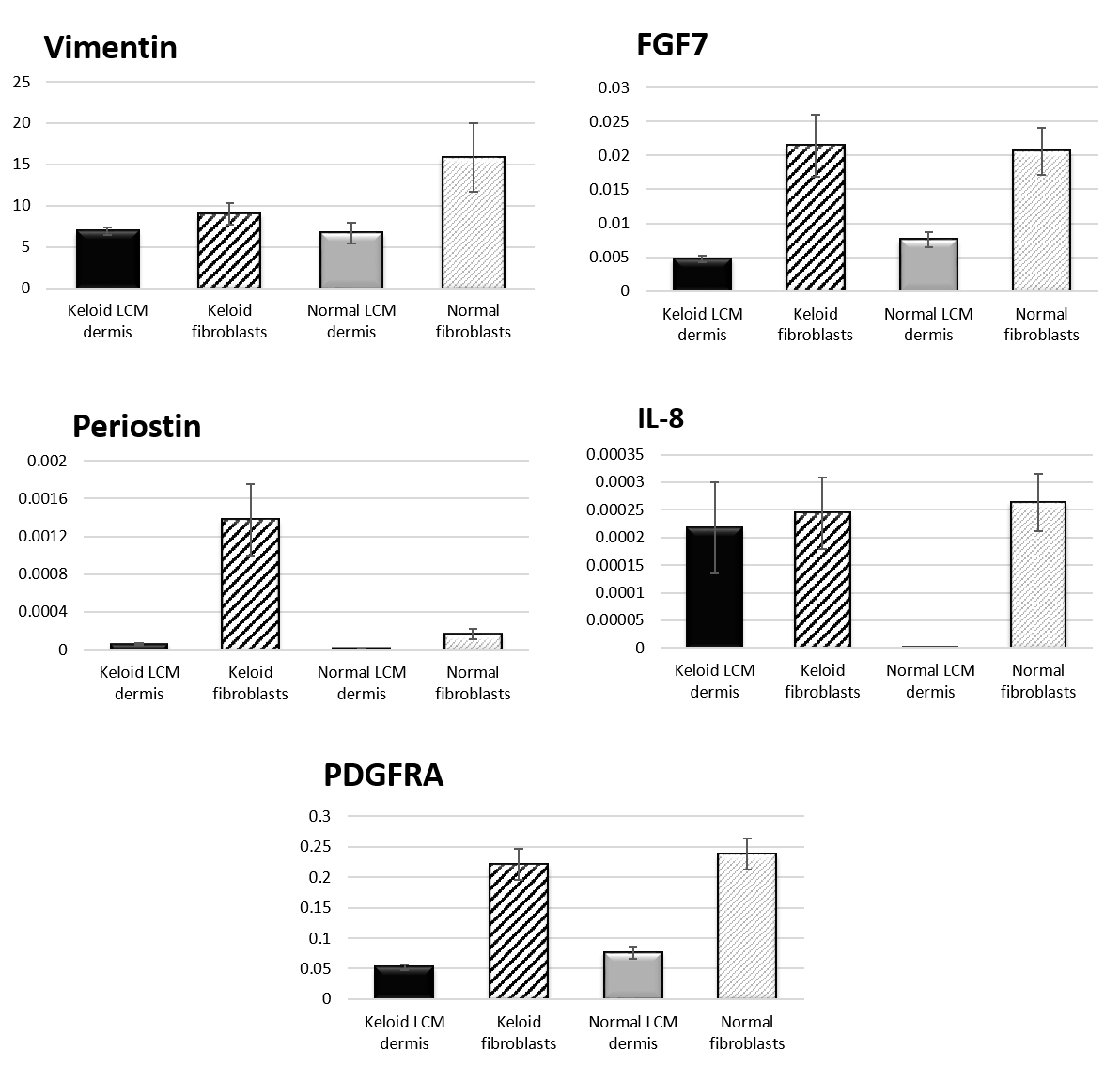


**B**


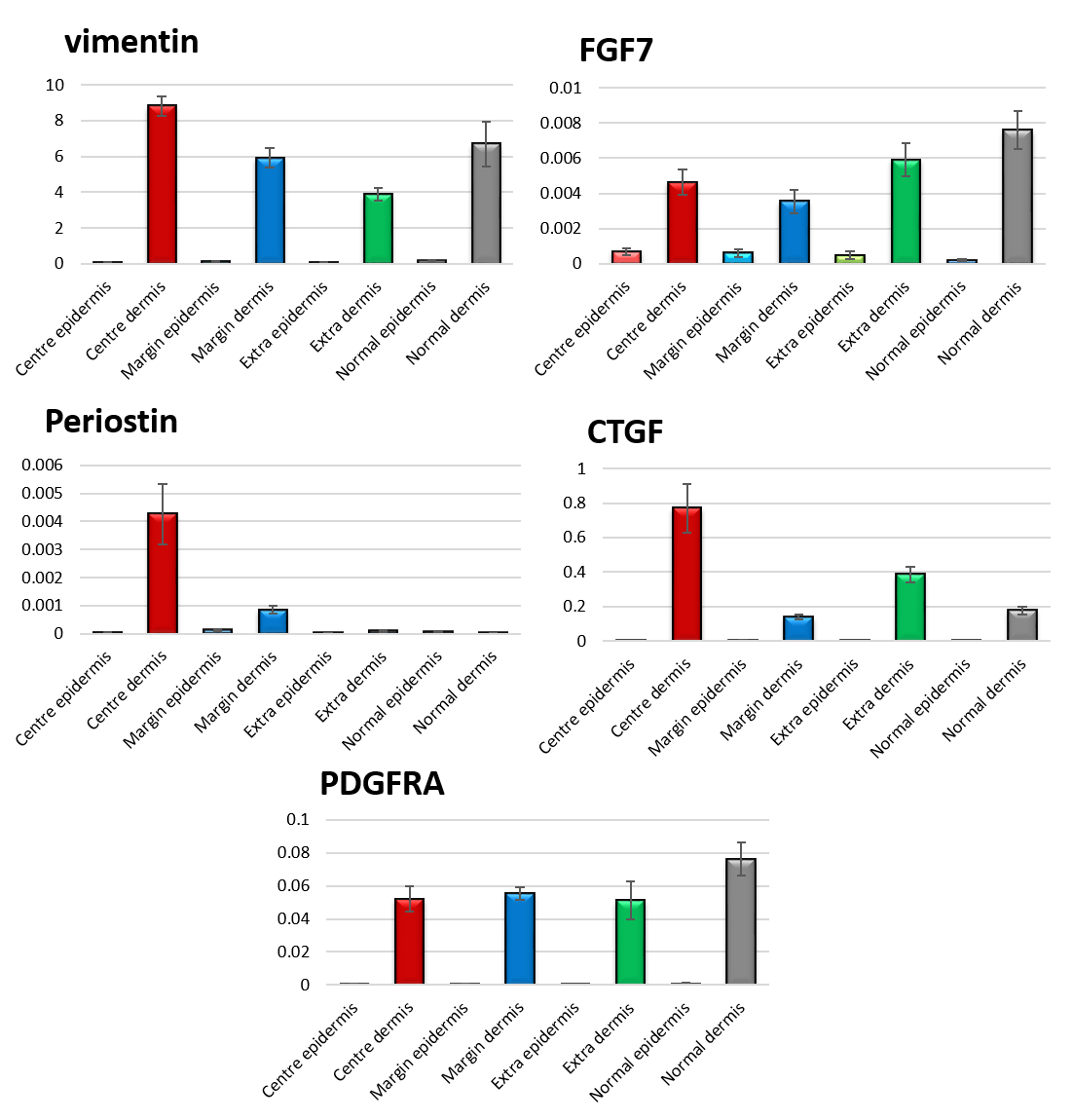

Supplement: S2 Fig — (DOCX) [file pone.0172955.s004.docx]
