# Supplementary figures and images for: Site-specific gene expression profiling as a novel strategy for unravelling keloid disease pathobiology
Source: PLoS One. 2017 Mar 3;12(3):e0172955. doi: 10.1371/journal.pone.0172955 (PMC5336271; doi:10.1371/journal.pone.0172955)

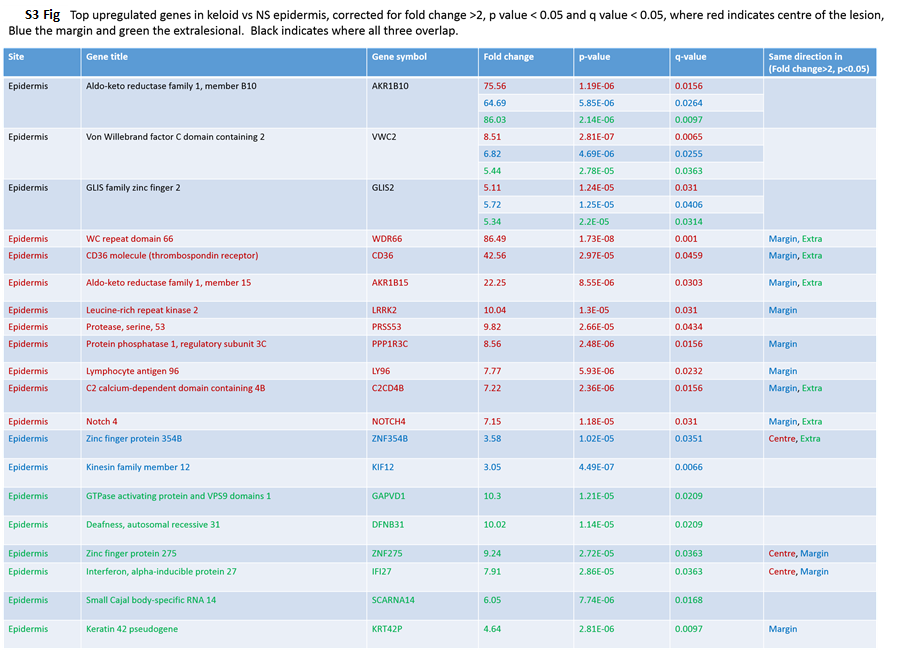

Supplement: S3 Fig — (DOCX) [file pone.0172955.s005.docx]

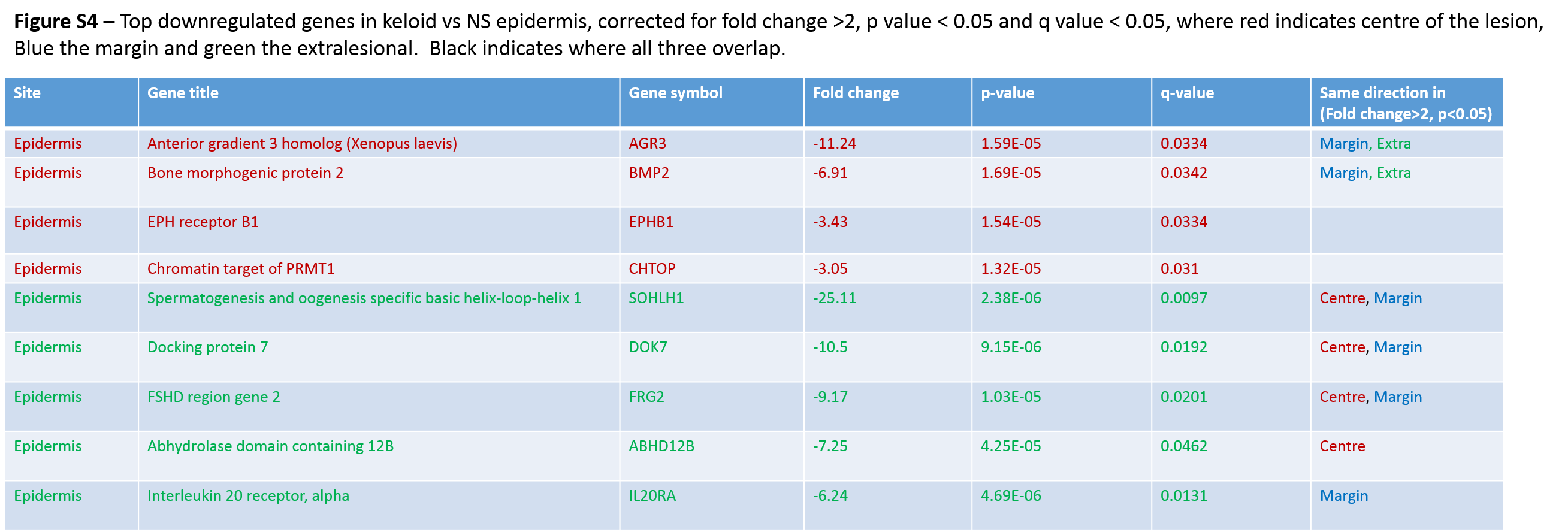


**S4 Fig**

Supplement: S4 Fig — (DOCX) [file pone.0172955.s006.docx]

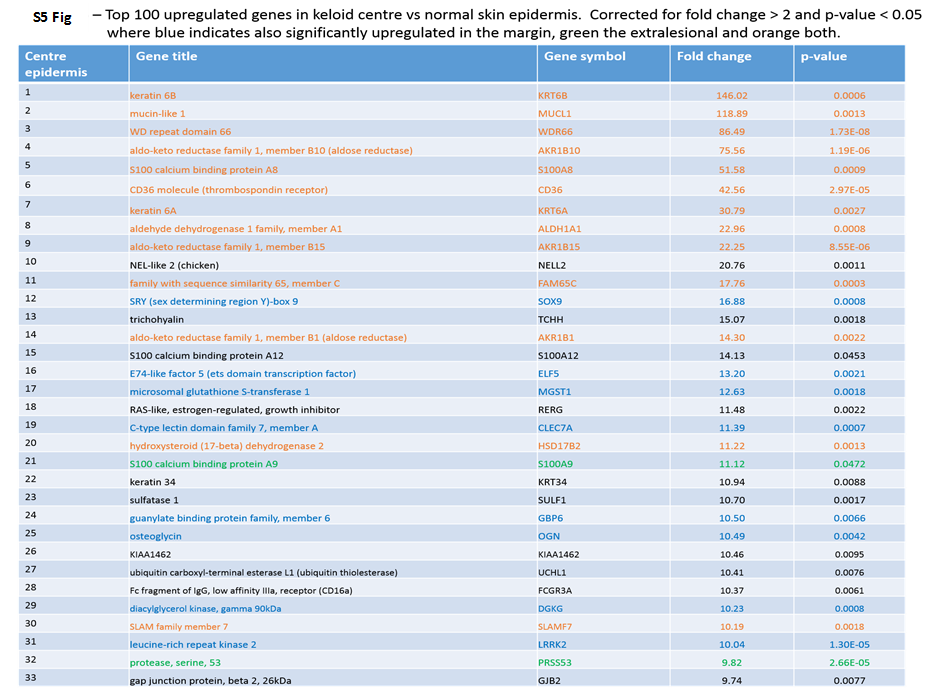


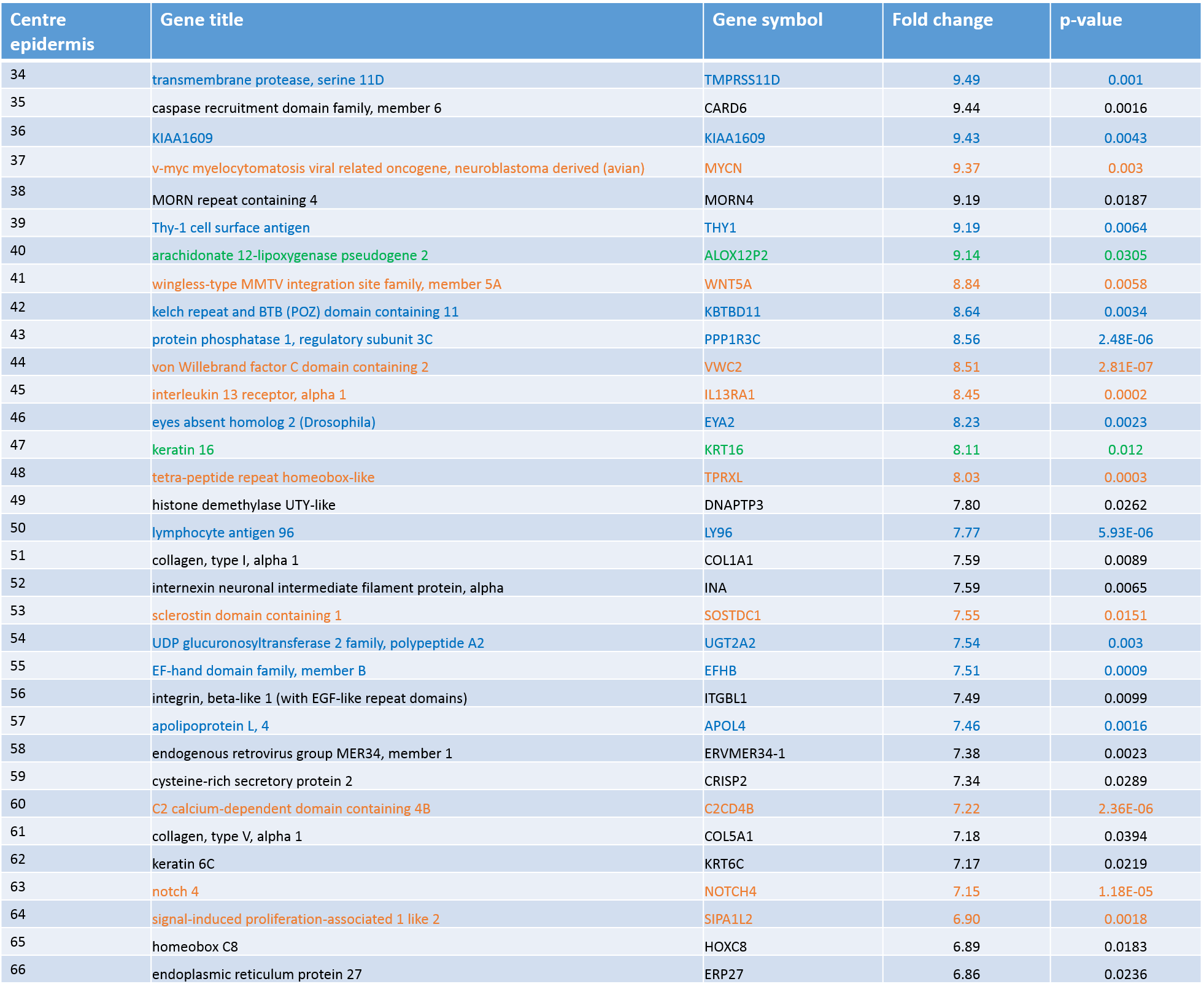


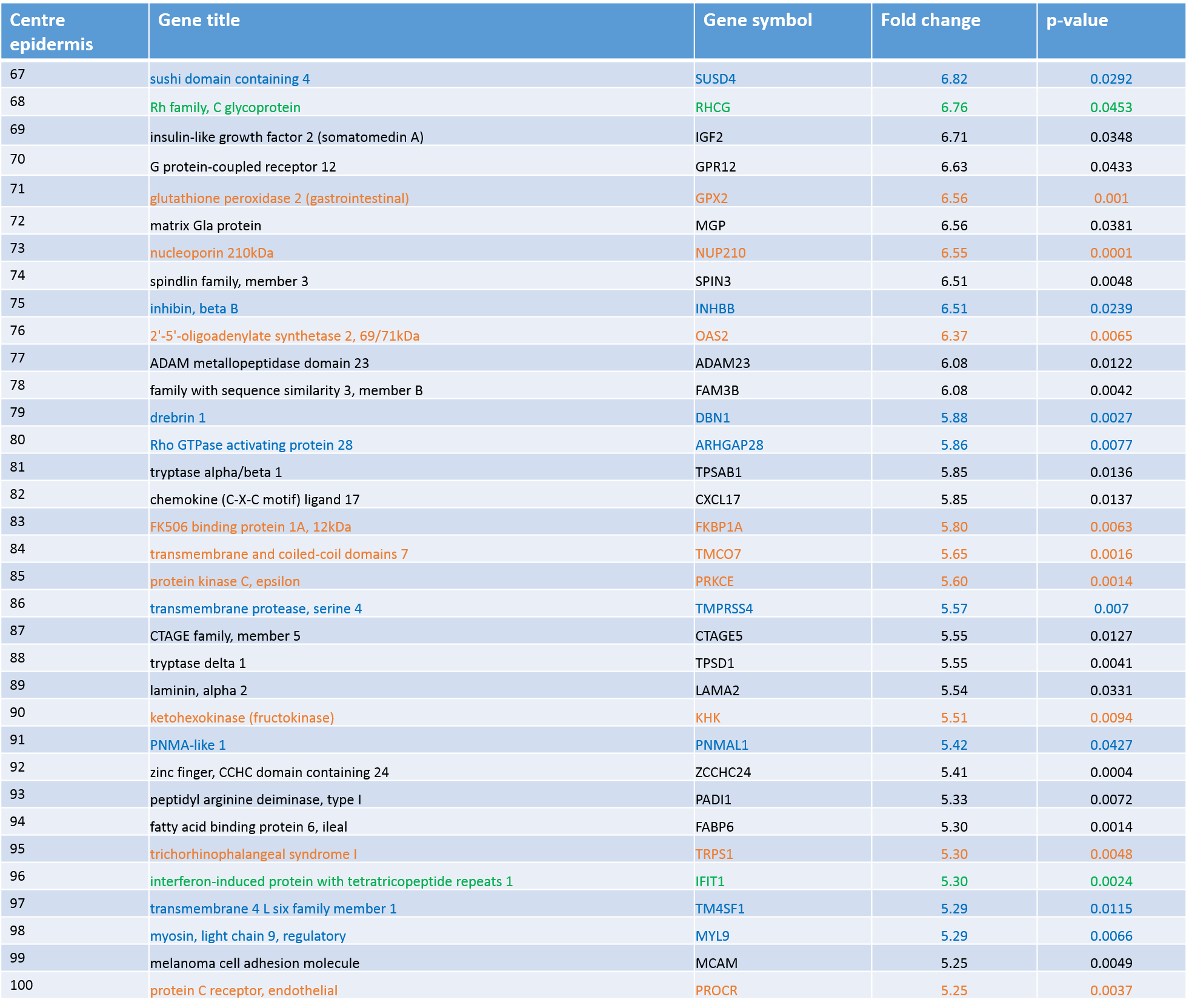

Supplement: S5 Fig — (DOCX) [file pone.0172955.s007.docx]

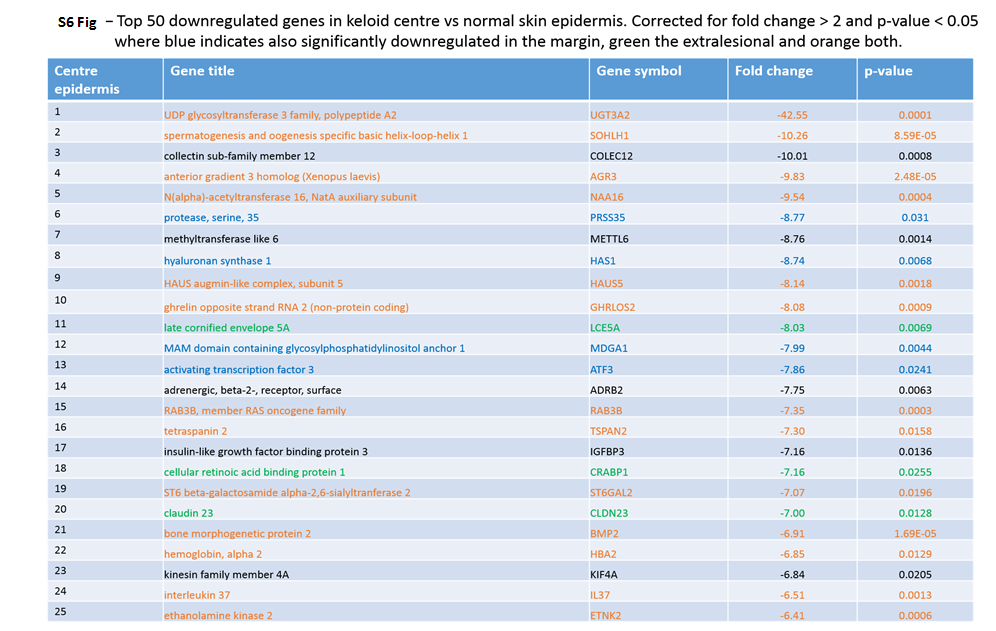


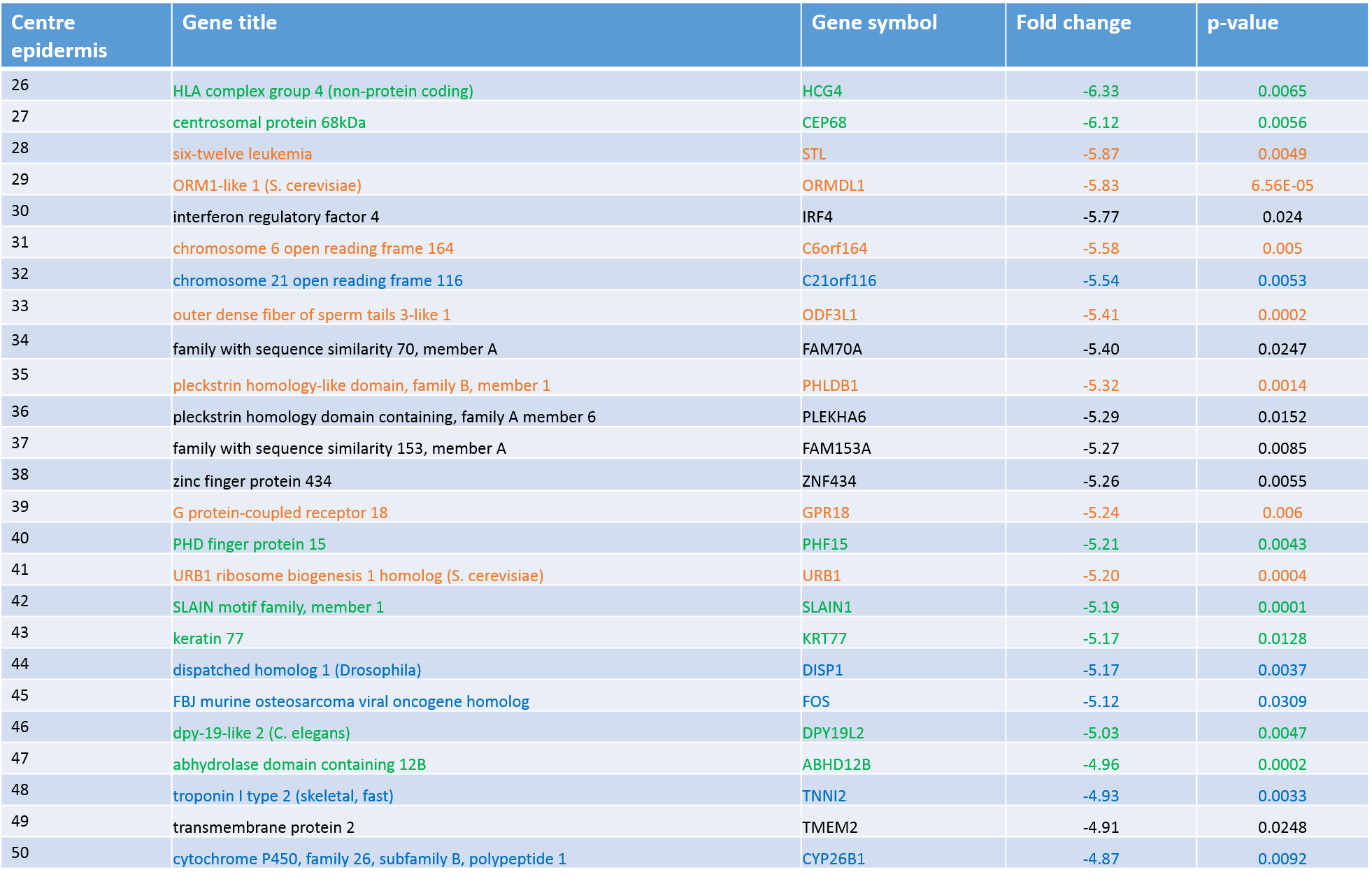

Supplement: S6 Fig — (DOCX) [file pone.0172955.s008.docx]

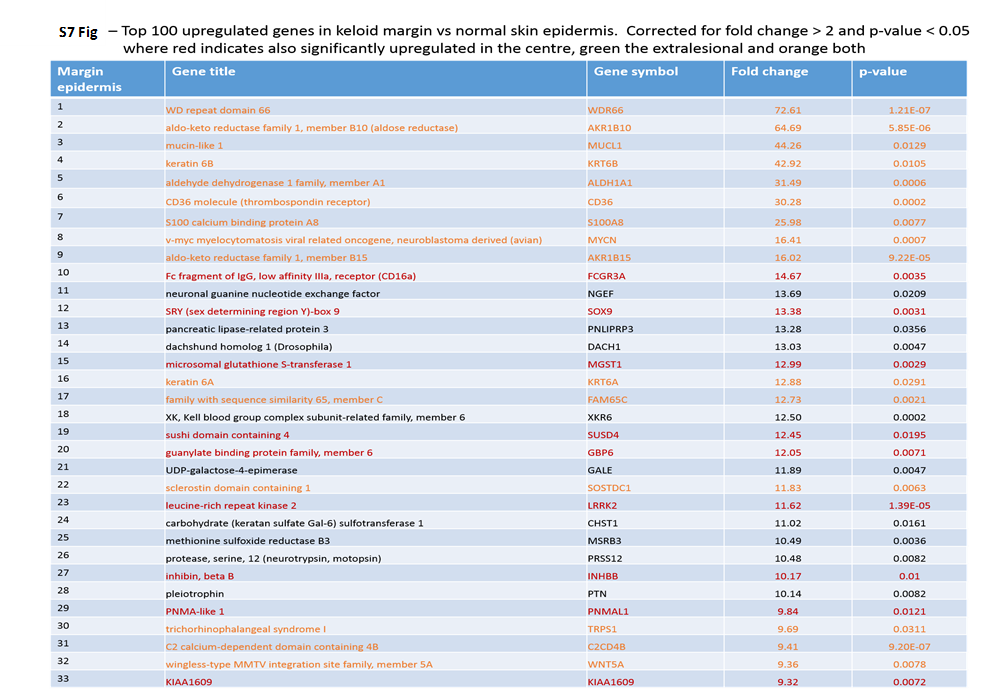


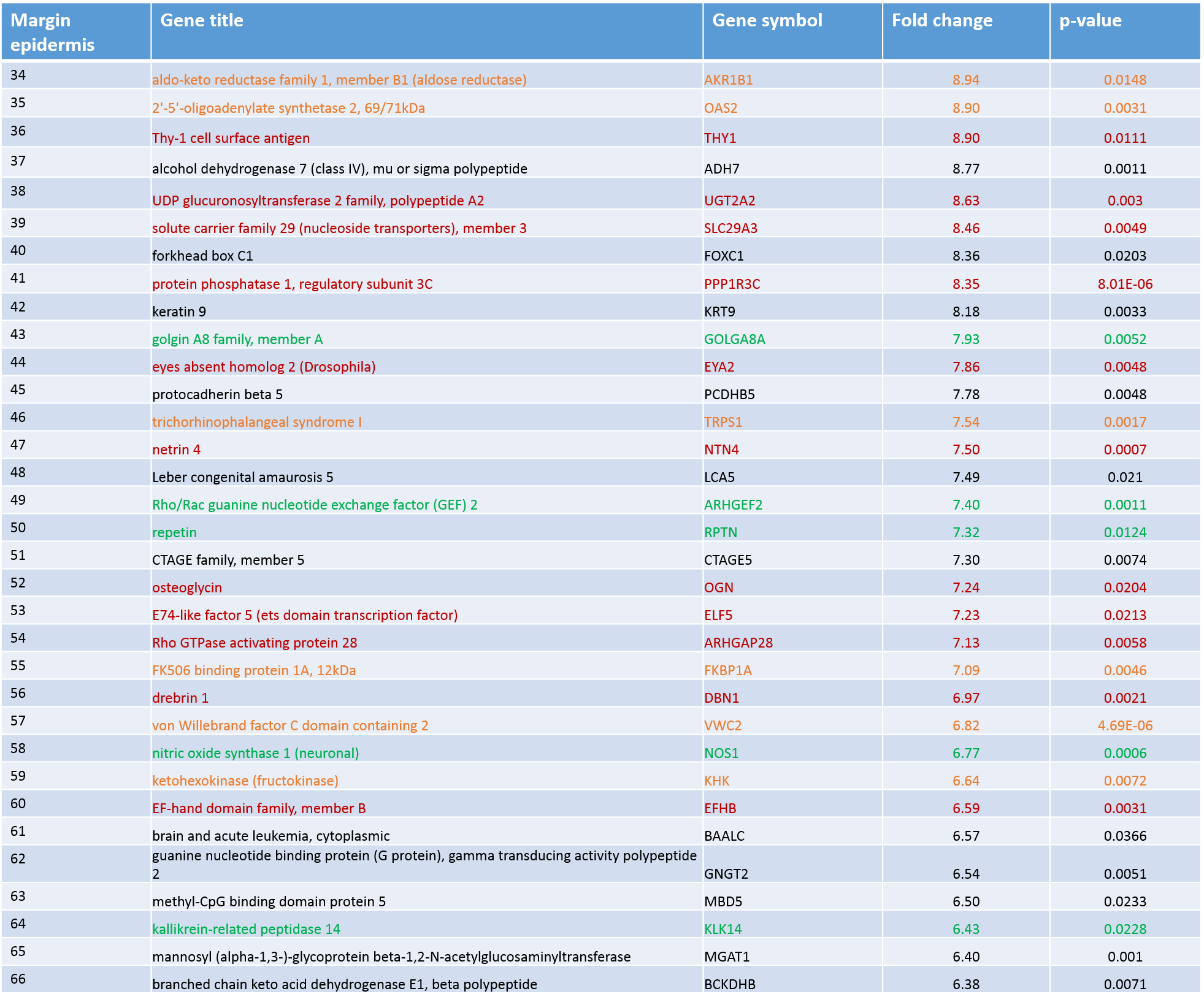


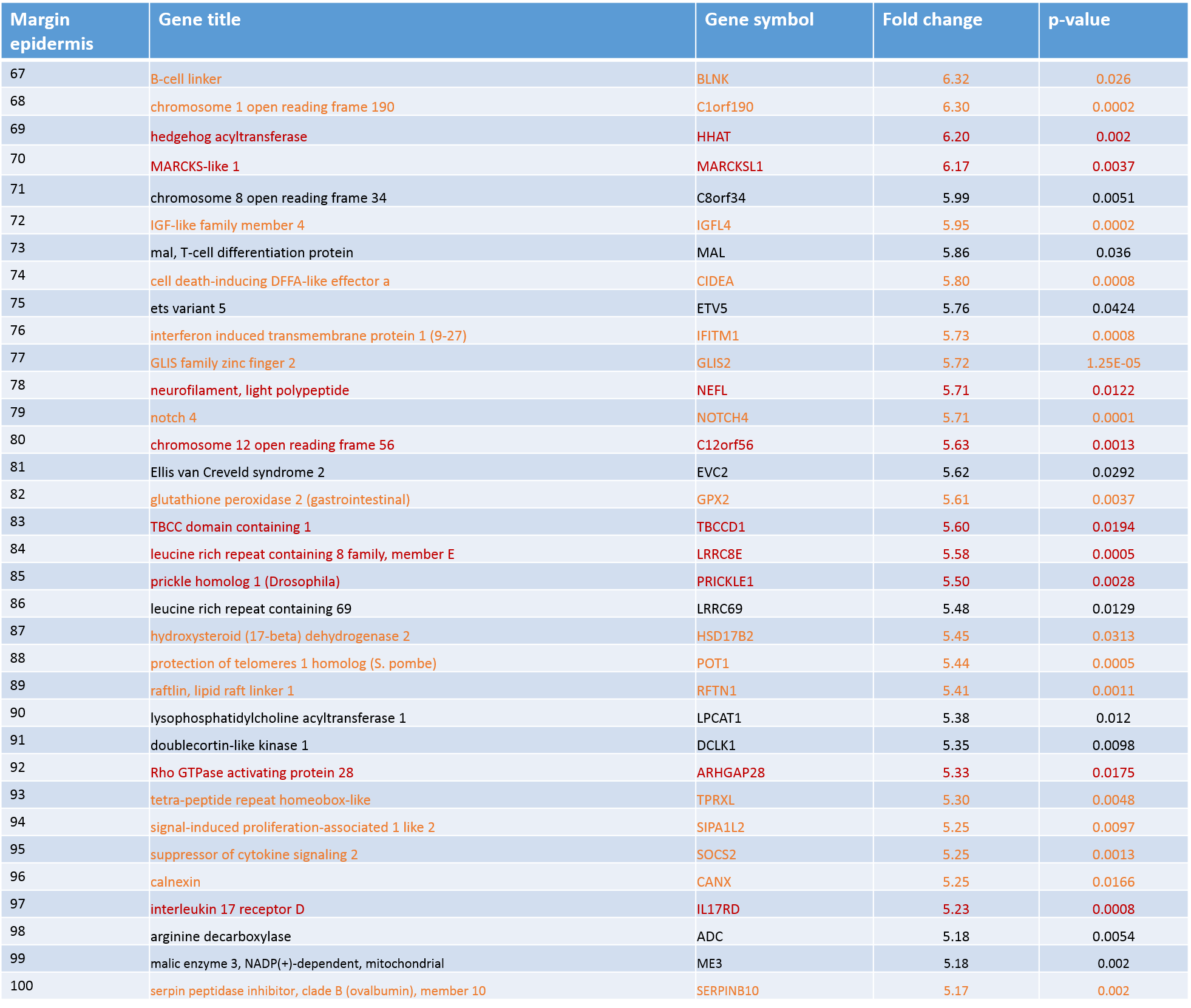

Supplement: S7 Fig — (DOCX) [file pone.0172955.s009.docx]

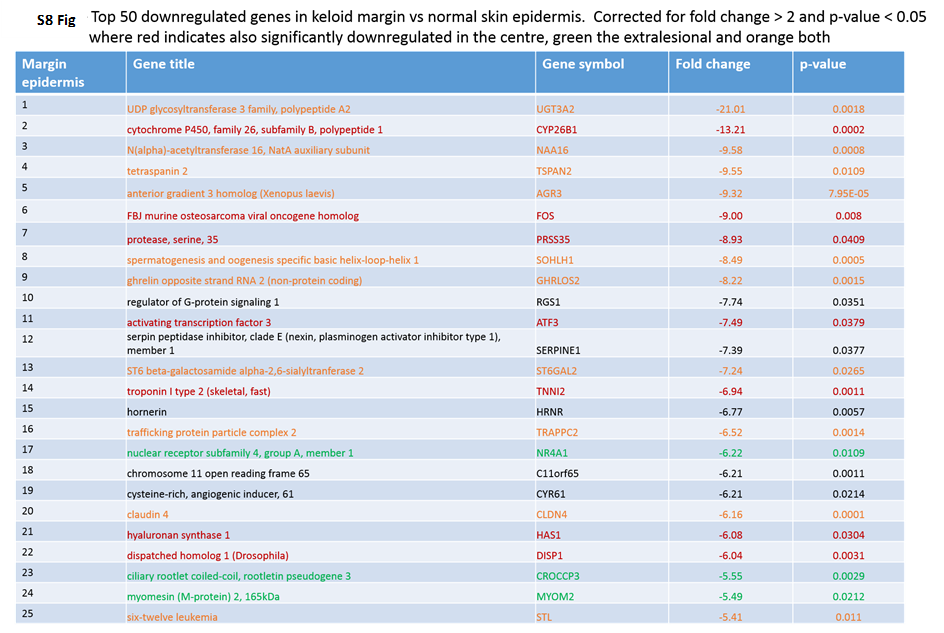


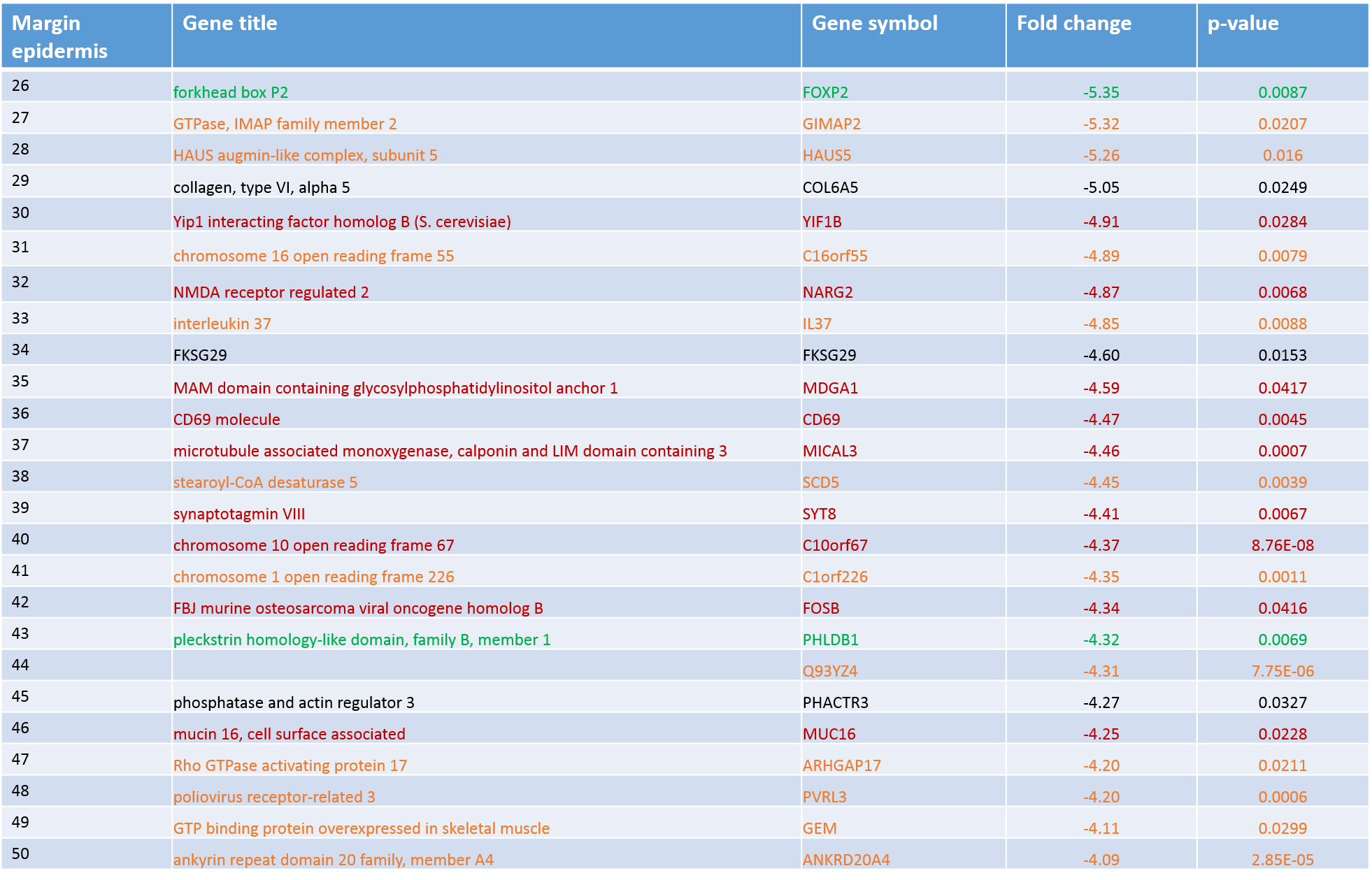

Supplement: S8 Fig — (DOCX) [file pone.0172955.s010.docx]

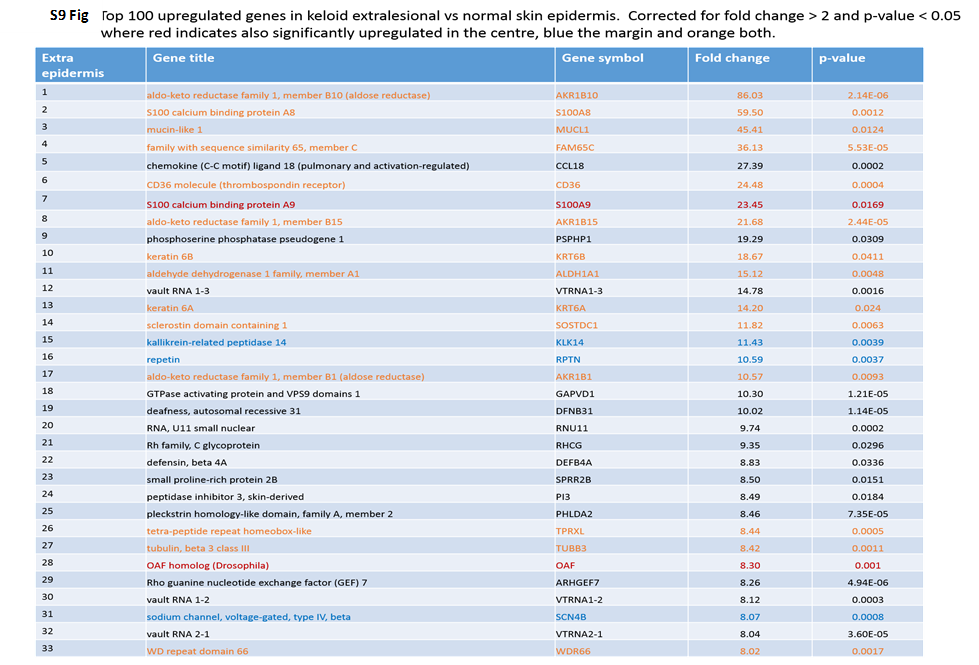


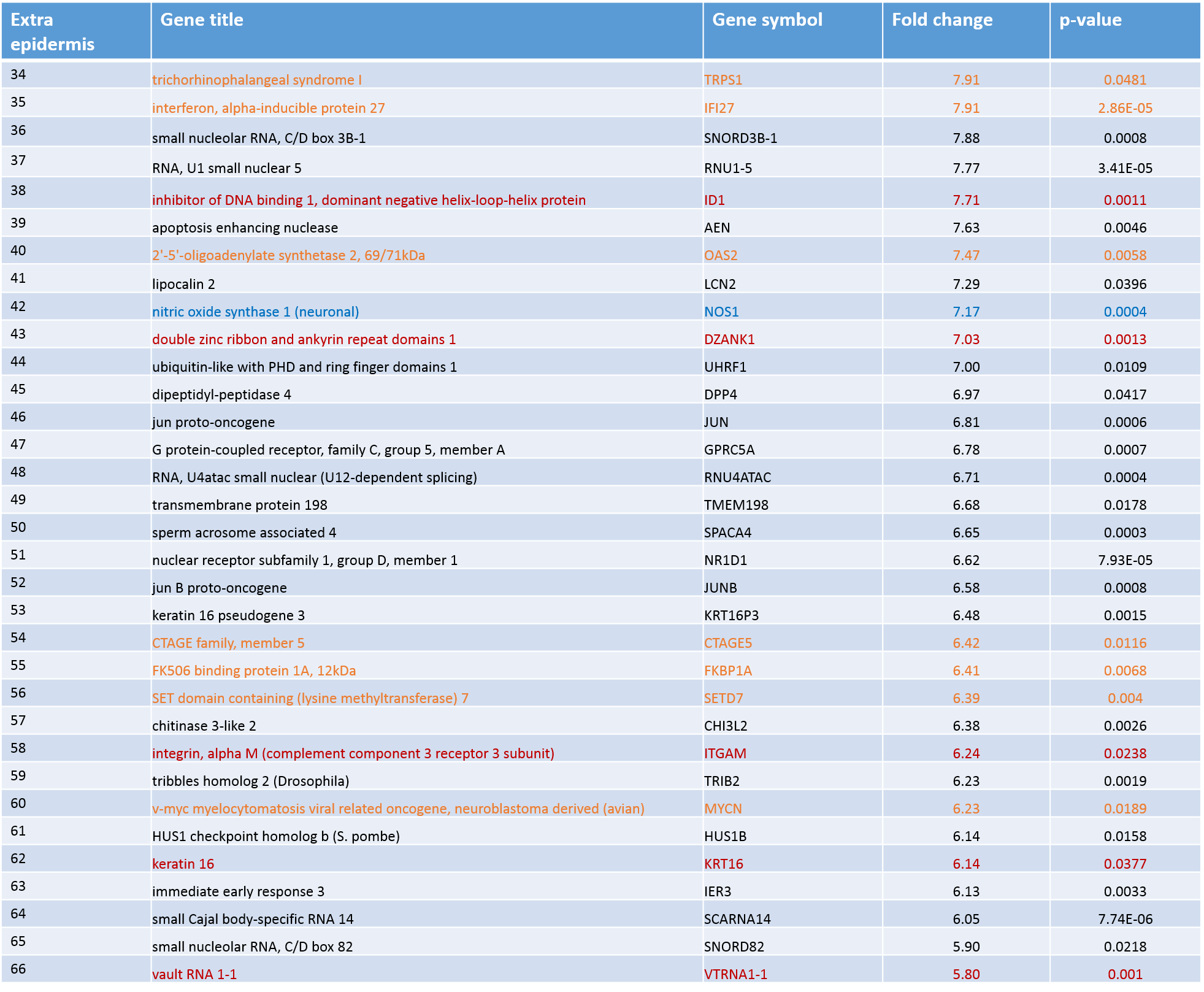


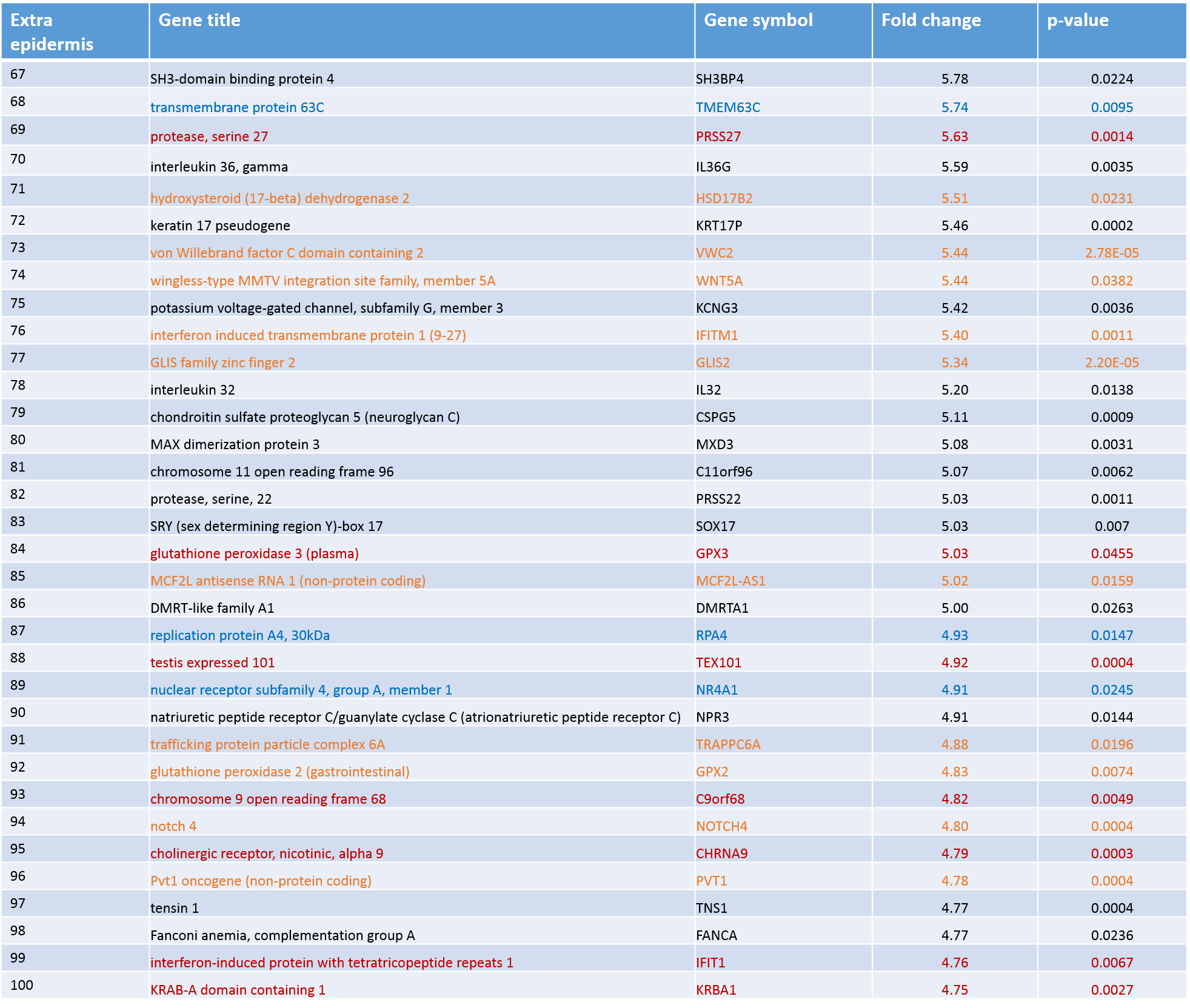

Supplement: S9 Fig — (DOCX) [file pone.0172955.s011.docx]

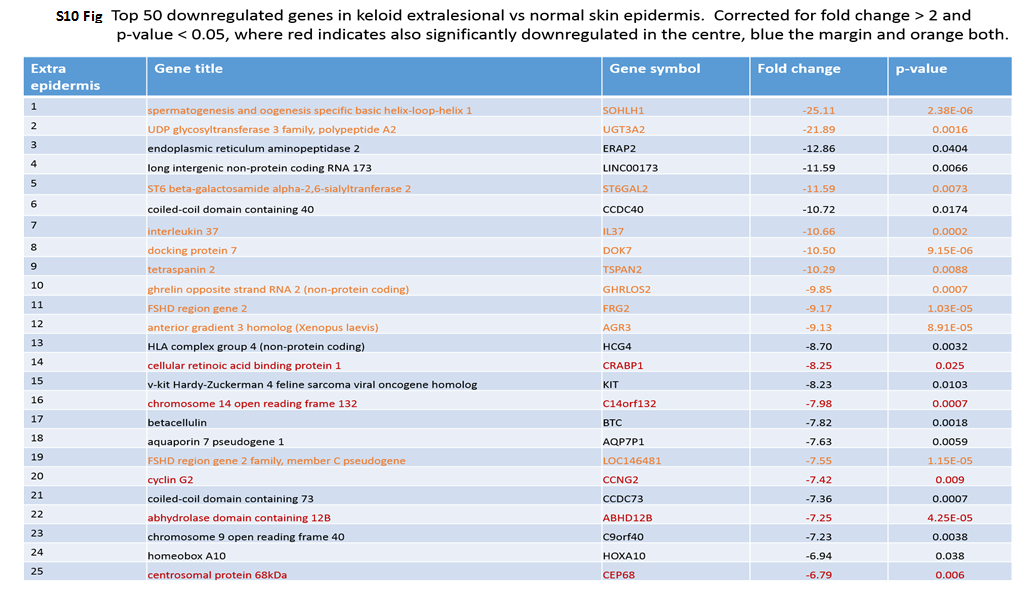


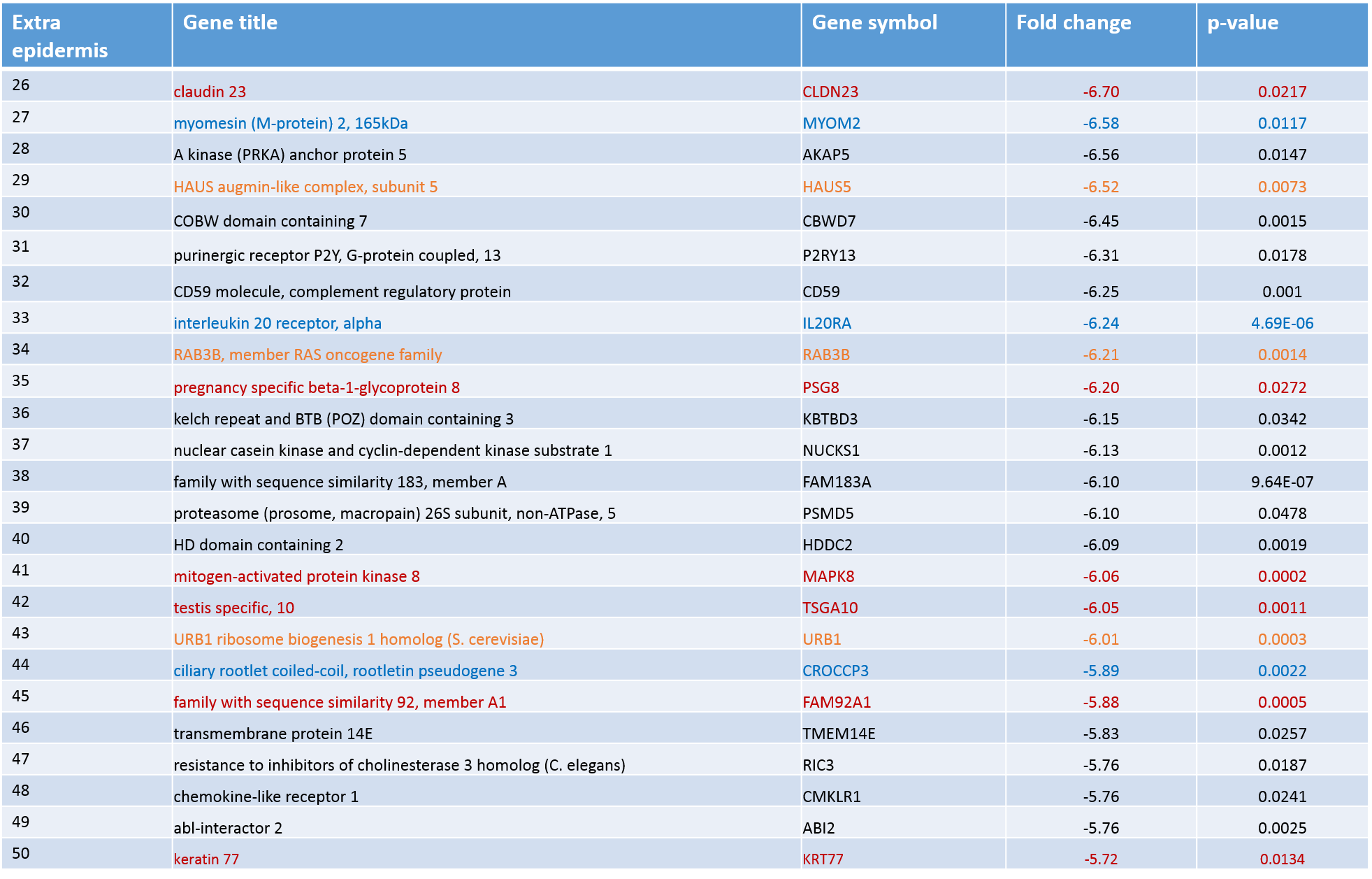

Supplement: S10 Fig — (DOCX) [file pone.0172955.s012.docx]

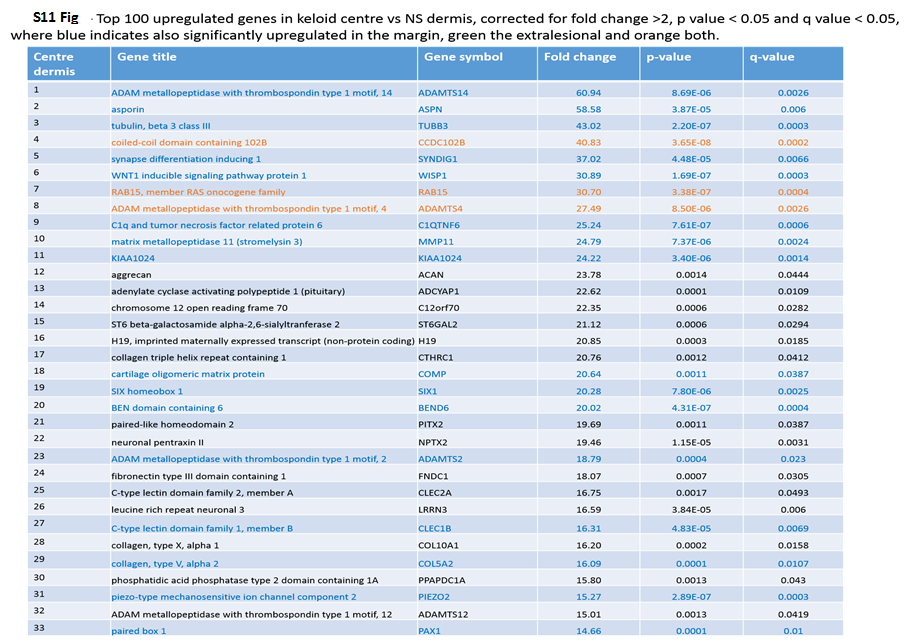


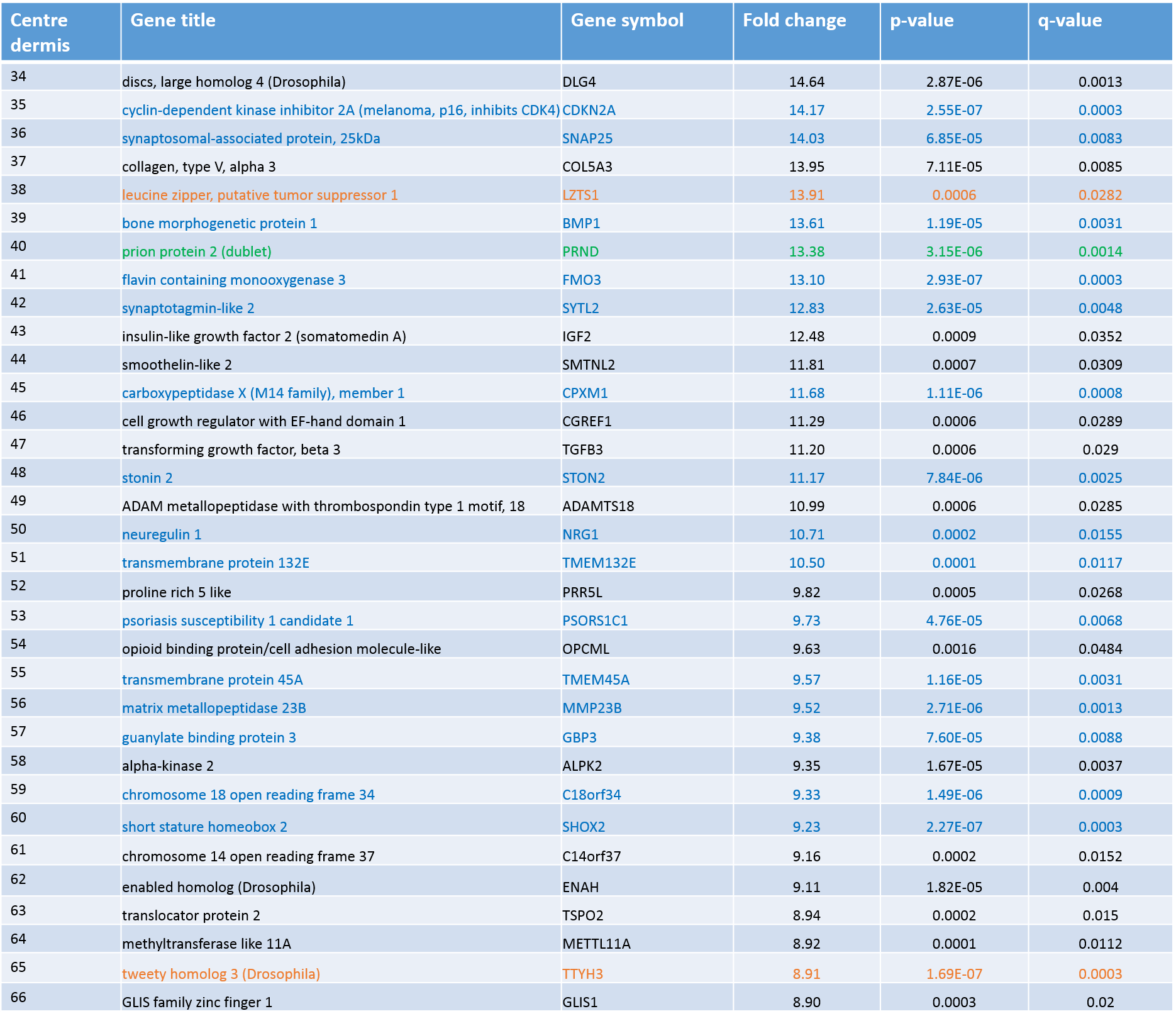


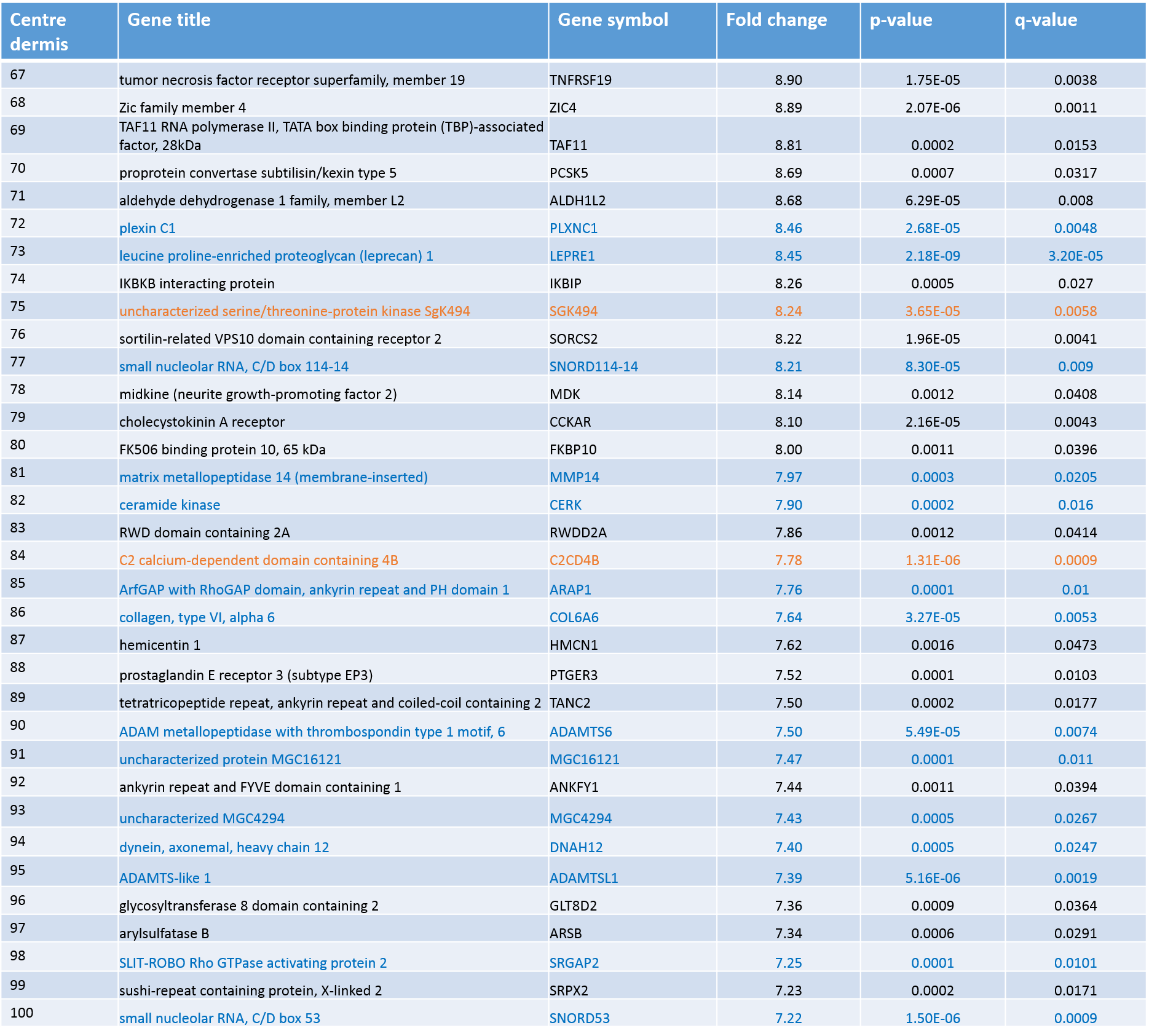

Supplement: S11 Fig — (DOCX) [file pone.0172955.s013.docx]

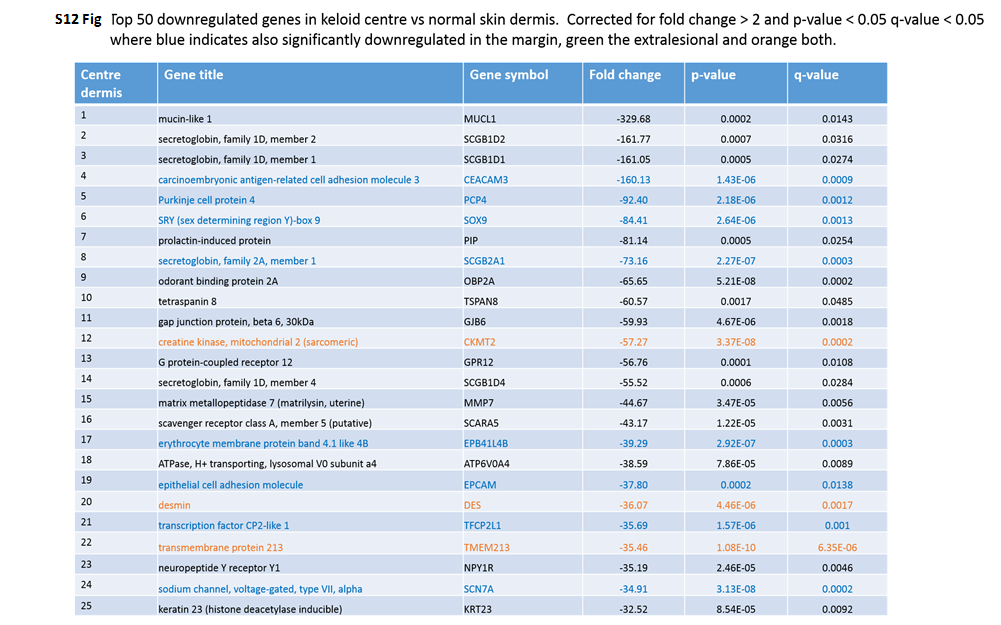


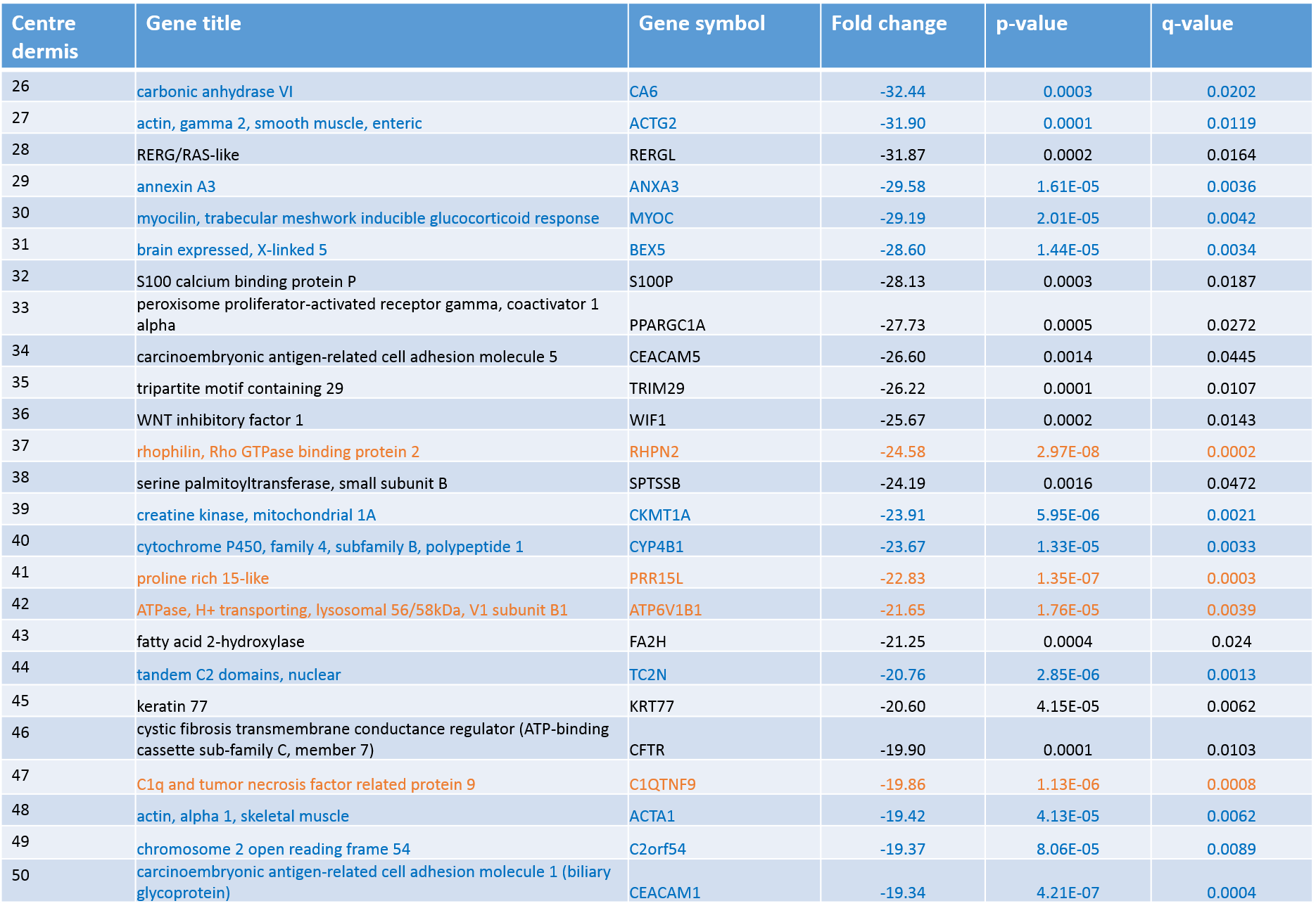

Supplement: S12 Fig — (DOCX) [file pone.0172955.s014.docx]

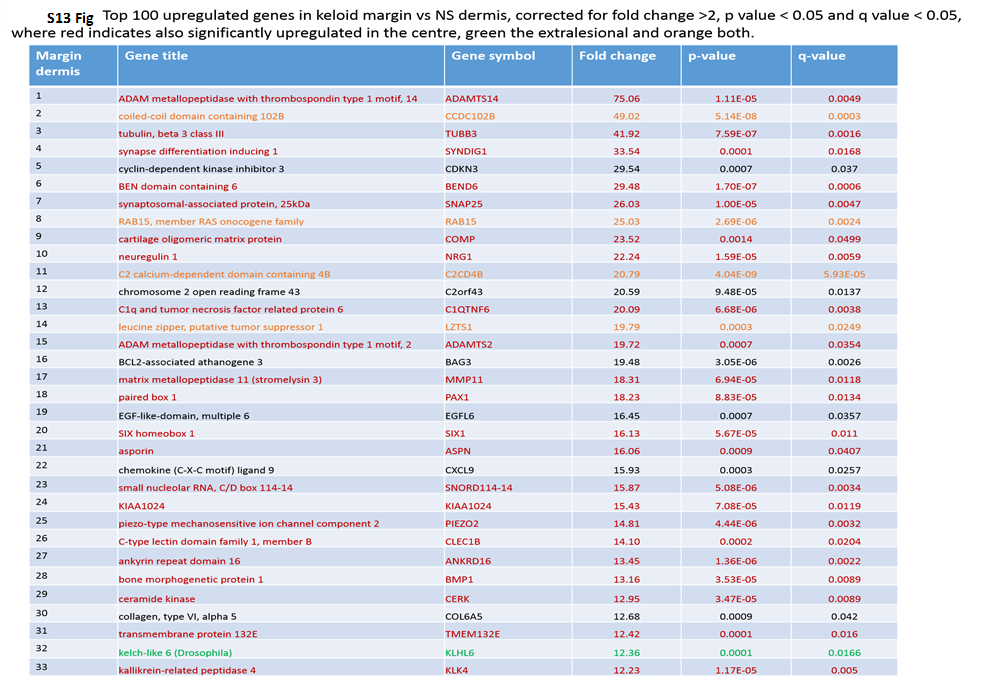


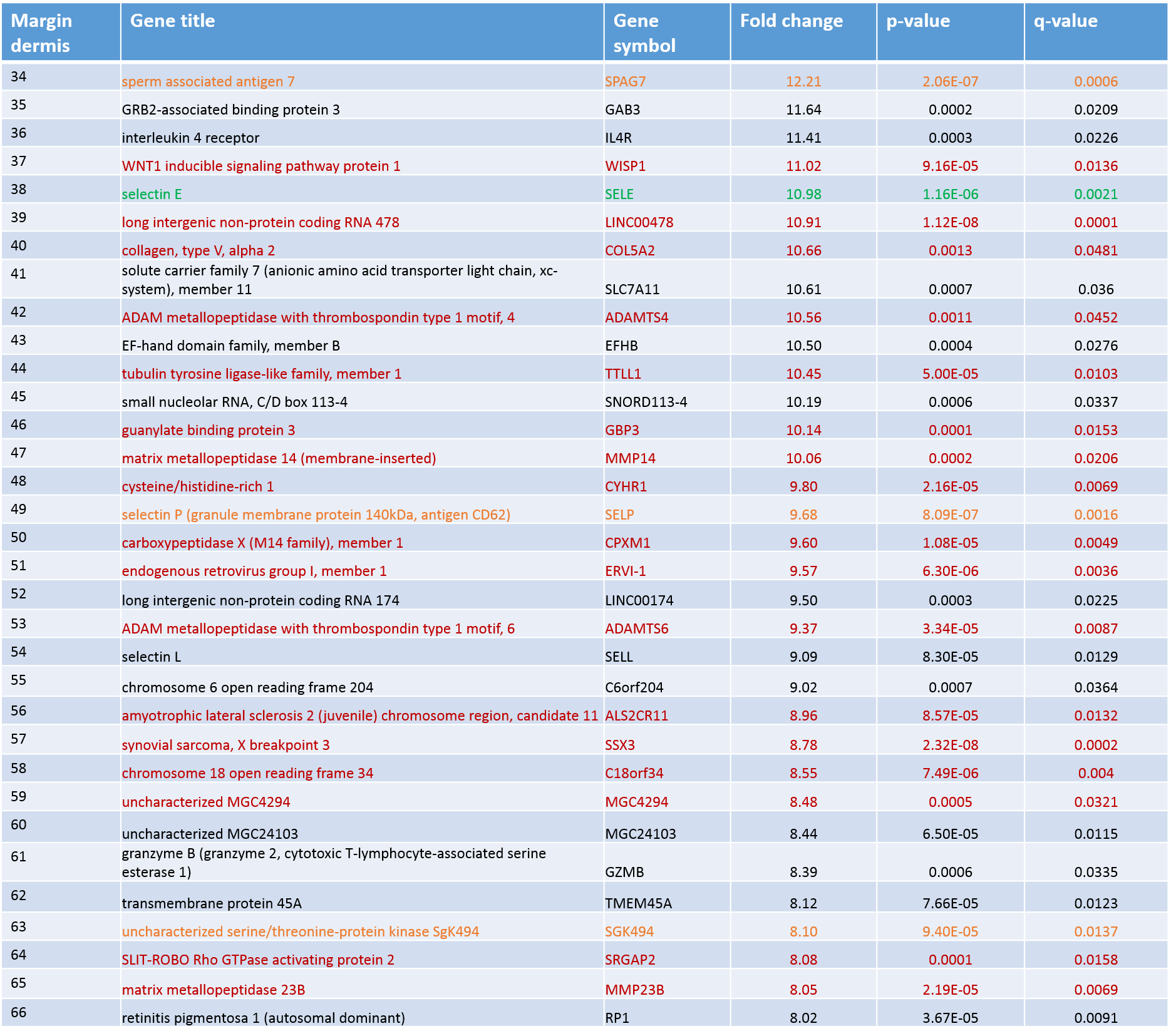


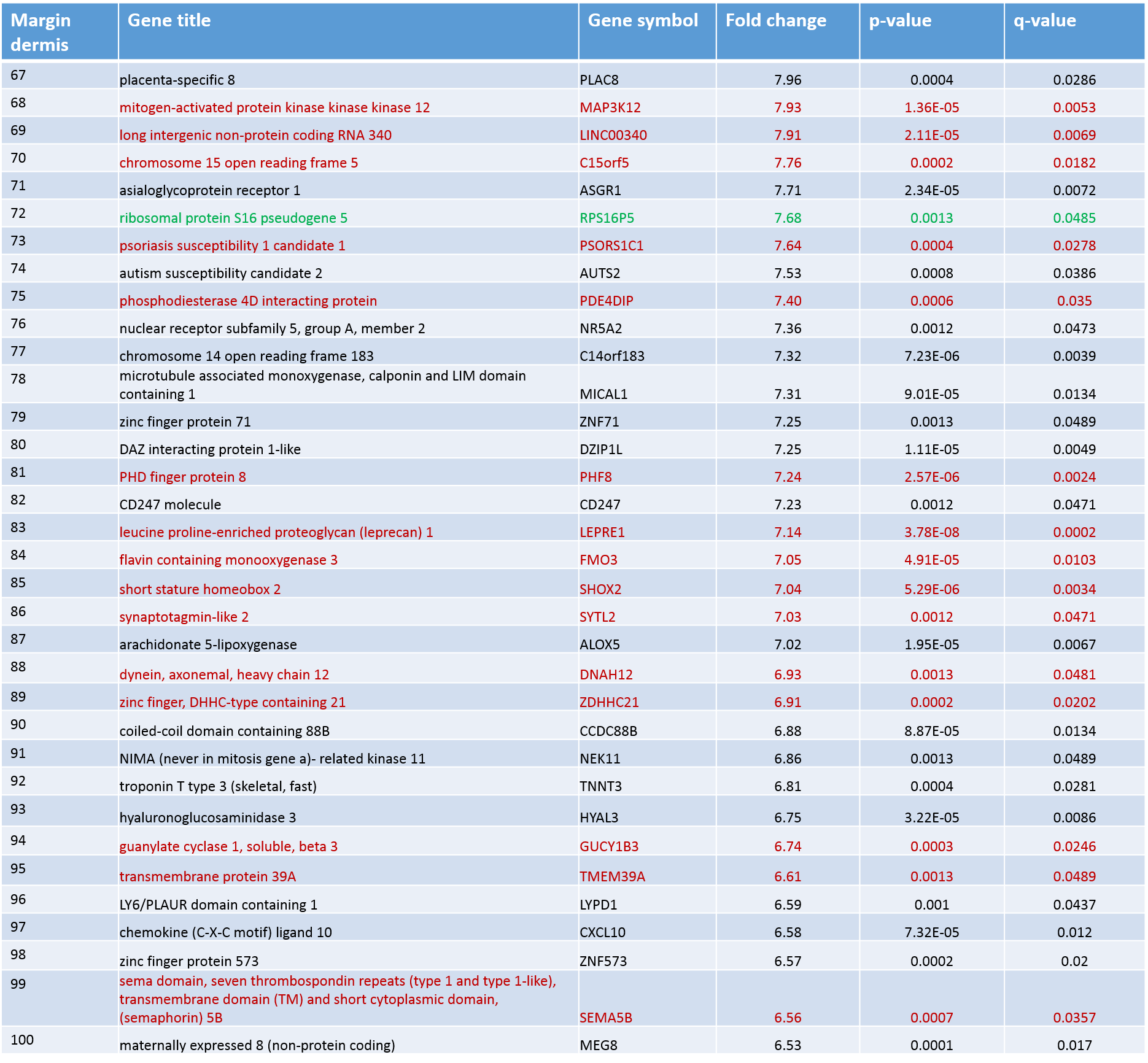

Supplement: S13 Fig — (DOCX) [file pone.0172955.s015.docx]

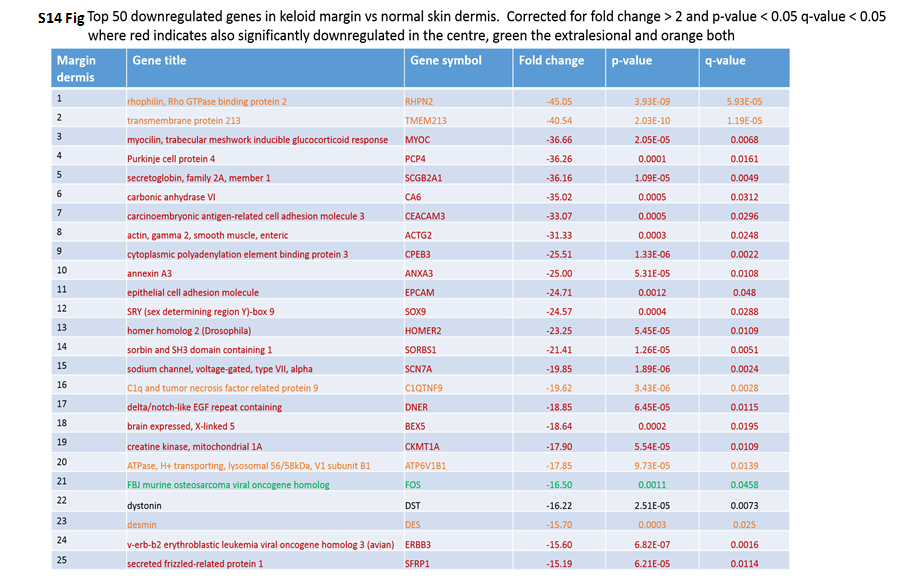


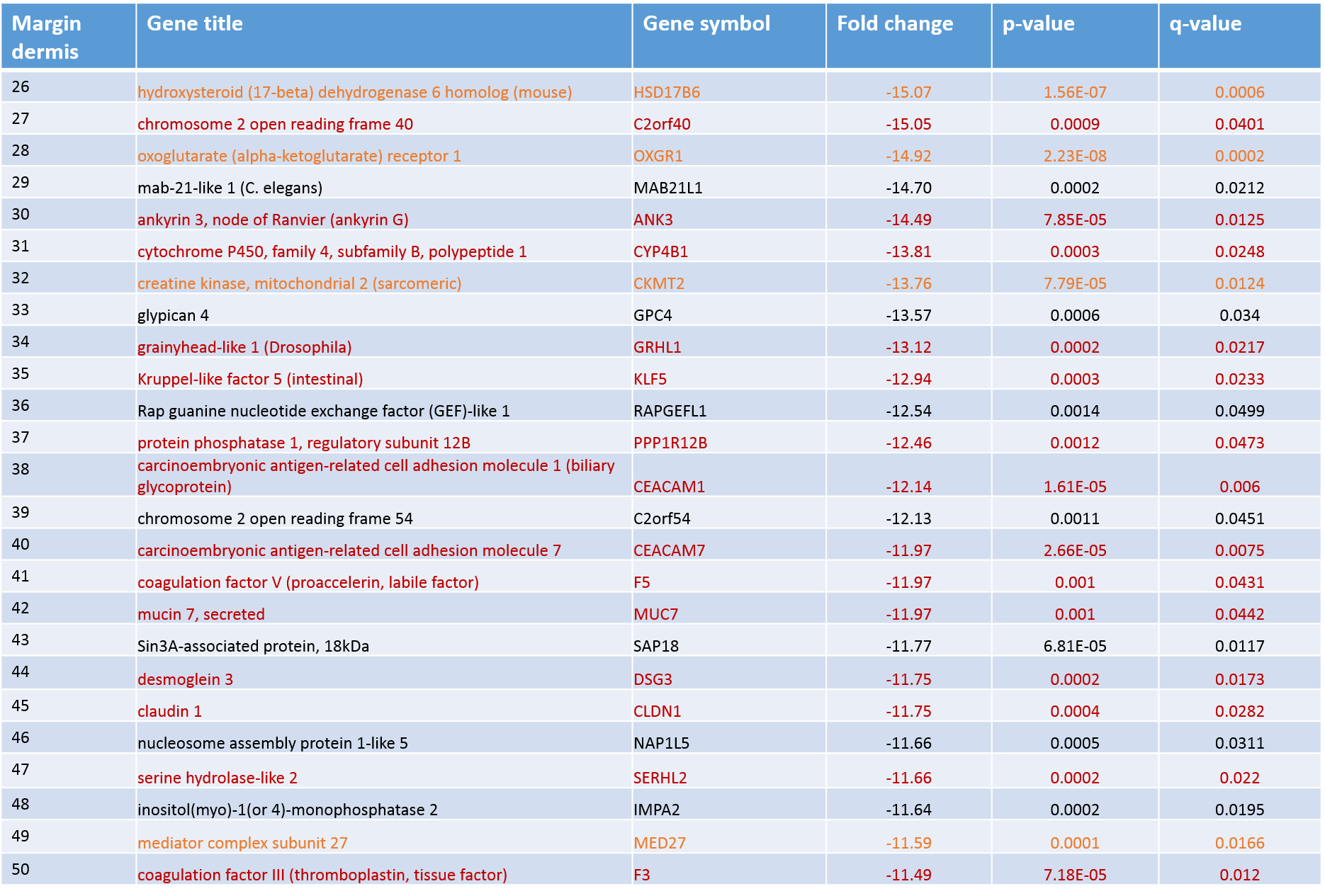

Supplement: S14 Fig — (DOCX) [file pone.0172955.s016.docx]

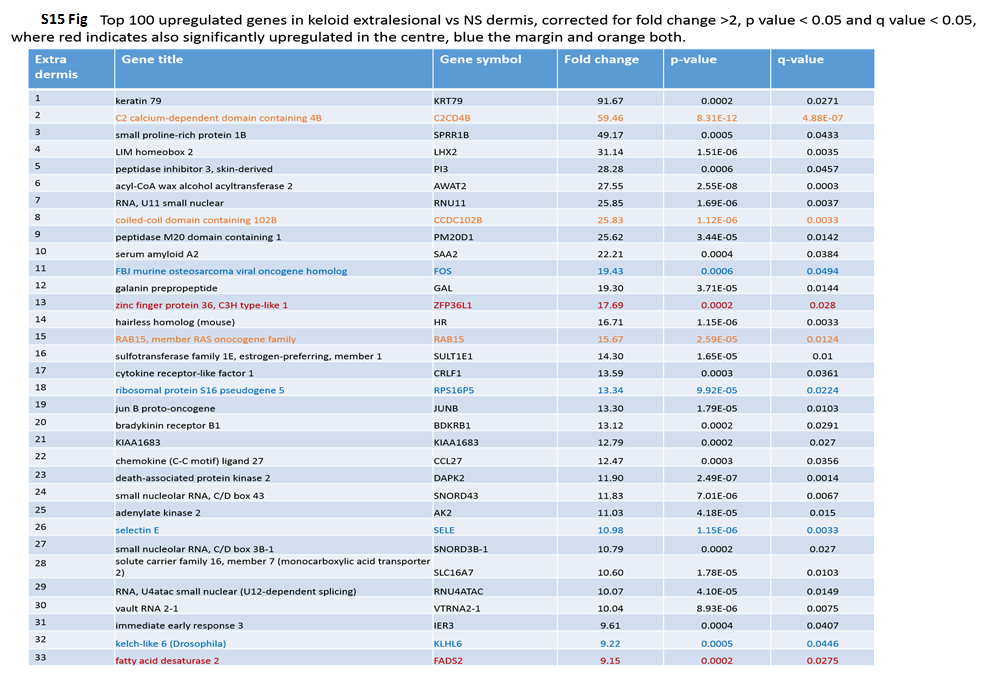


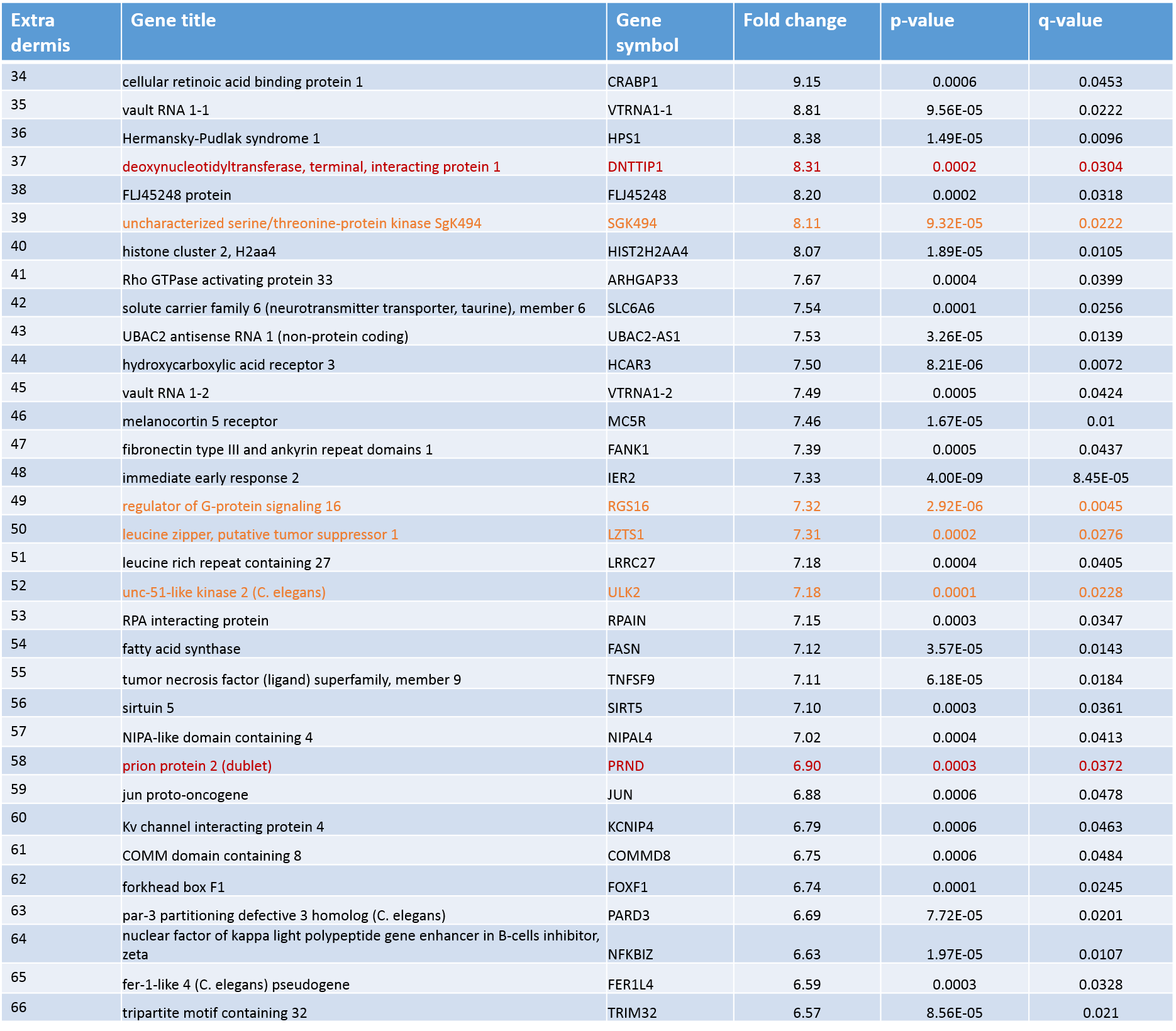


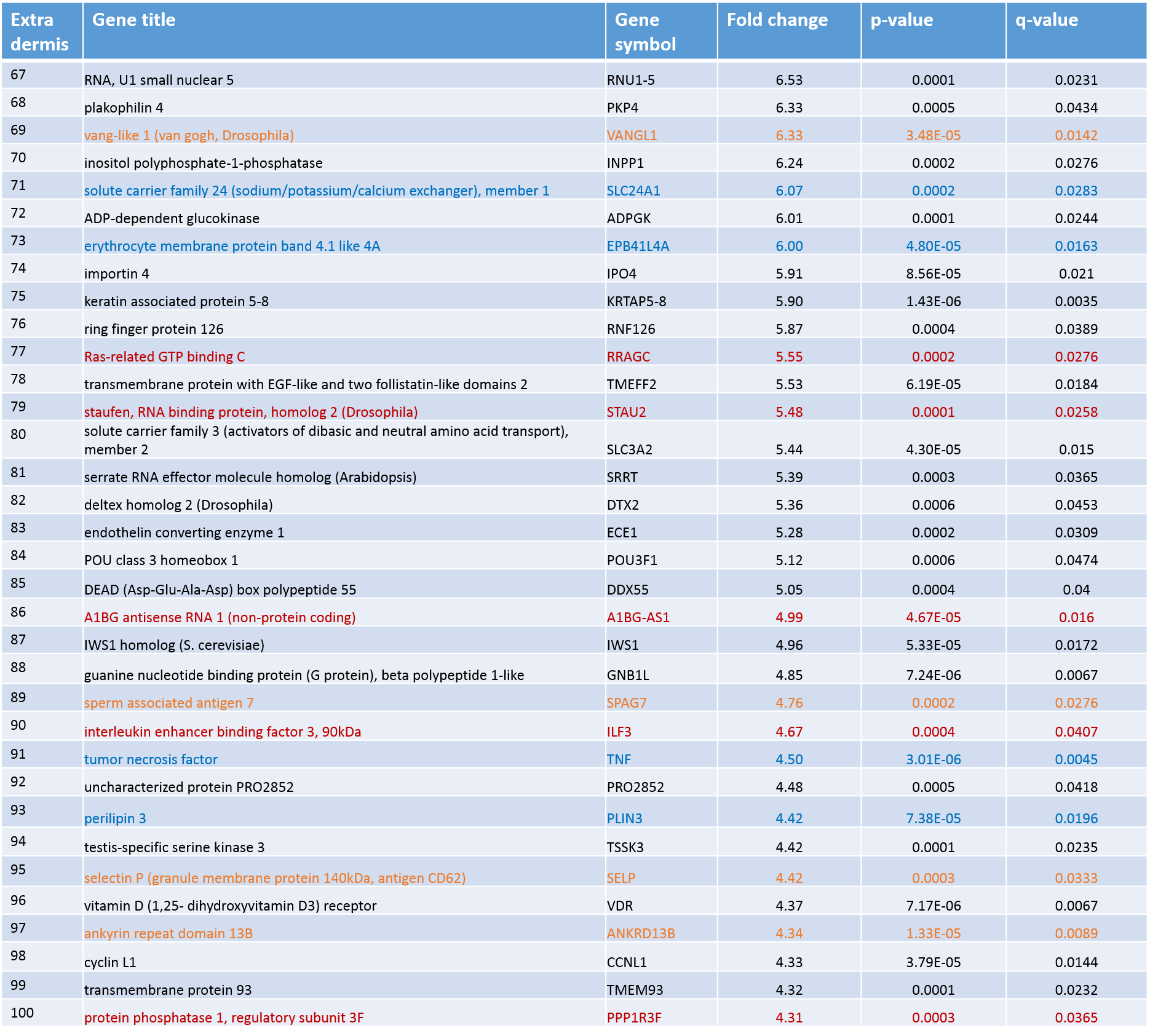

Supplement: S15 Fig — (DOCX) [file pone.0172955.s017.docx]

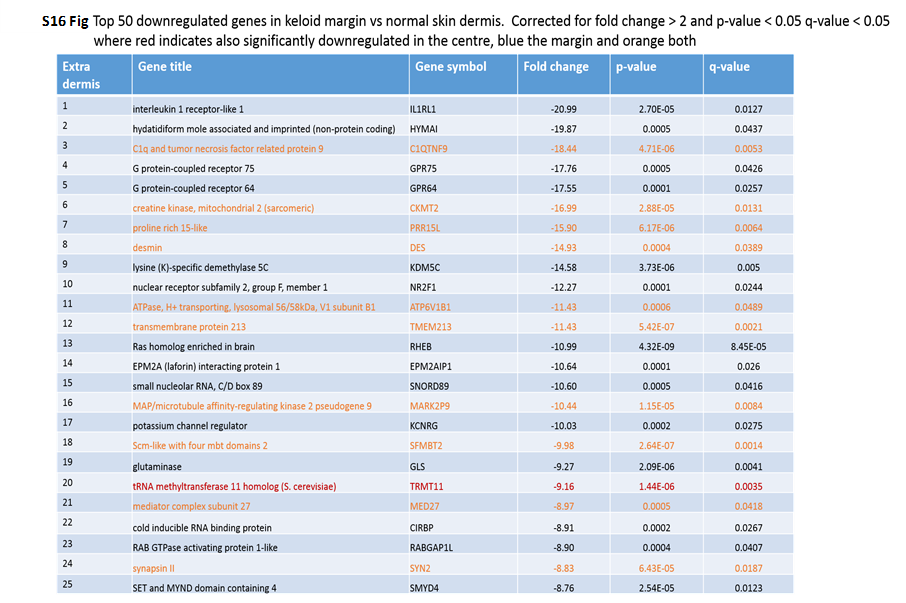


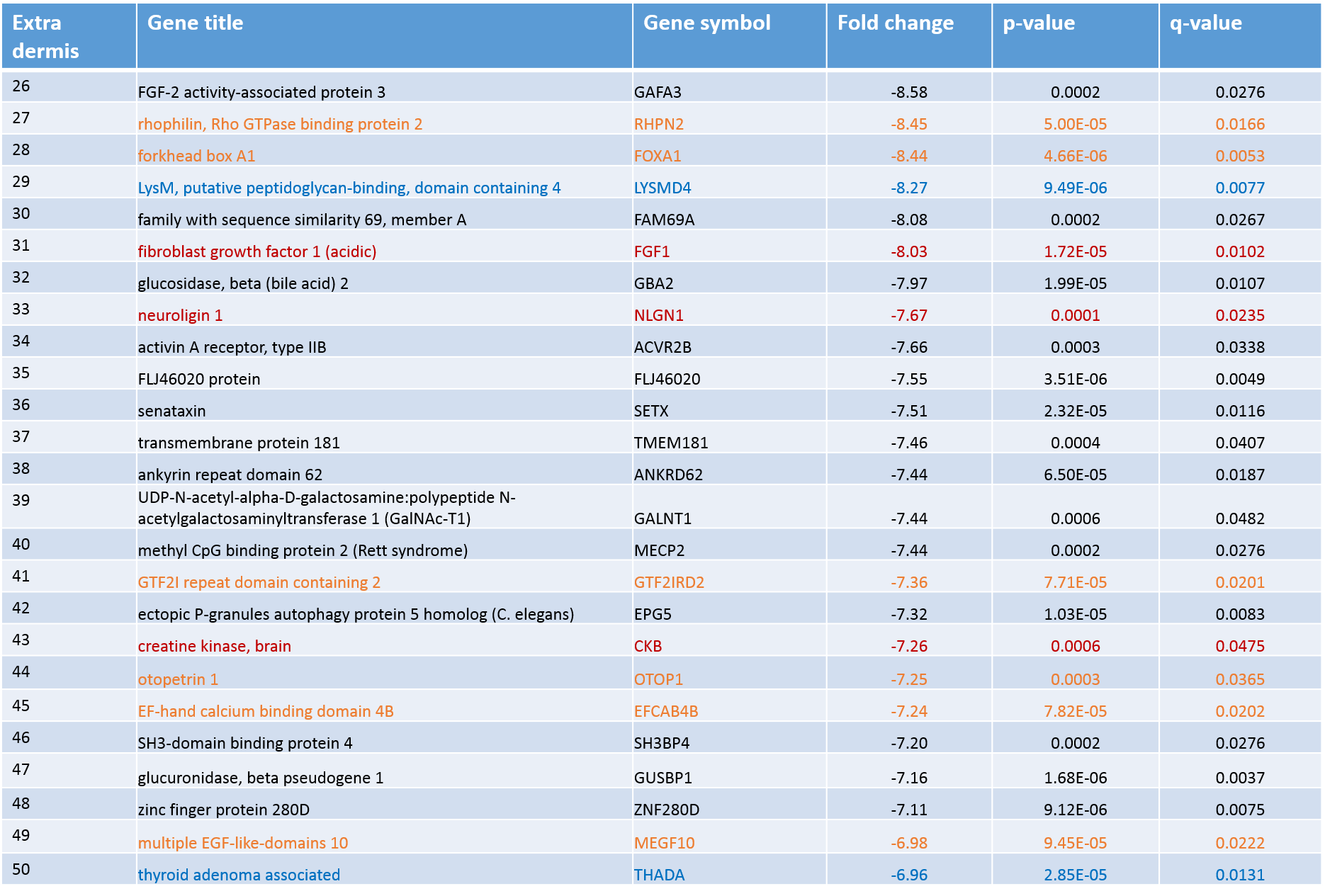

Supplement: S16 Fig — (DOCX) [file pone.0172955.s018.docx]
